# Causal associations between gut microbiota and cutaneous melanoma: a Mendelian randomization study

**DOI:** 10.3389/fmicb.2024.1339621

**Published:** 2024-04-08

**Authors:** Yan-Qiu Bao, Ying Zhang, Zhou-Na Li

**Affiliations:** ^1^Department of Medical Research Center, Shaoxing People’s Hospital, Zhejiang University School of Medicine, Shaoxing, Zhejiang, China; ^2^Department of Dermatology, Shaoxing People’s Hospital, Shaoxing, Zhejiang, China; ^3^Department of Dermatology, Affiliated Hospital of Yanbian University, Yanji, Jilin, China

**Keywords:** gut microbiota, cutaneous melanoma, Mendelian randomization, causal relationship, genetics

## Abstract

**Background:**

Cutaneous melanoma (CM) of the skin stands as the leading cause of mortality among skin cancer-related deaths. Despite the successes achieved with novel therapies such as immunotherapy and targeted therapy, their efficacy remains limited, necessitating further exploration of new treatment modalities. The gut microbiota and CM may be linked, as indicated by a growing body of preclinical and observational research. Nevertheless, the exact correlation between the intestinal microbiota and CM remains to be determined. Therefore, this study aims to assess the potential causal relationship between the gut microbiota and CM.

**Methods:**

The study utilized exposure data obtained from the MiBioGen consortium’s microbiome GWAS, which included a total of 18,340 samples gathered from 24 population-based cohorts. Data at the summary level for CM were acquired from the UK Biobank investigation. The main analytical strategy utilized in this research was the inverse variance weighted (IVW) technique, supported by quality assurance measures like the weighted median model, MR-Egger, simple model, and weighted model approaches. The Cochran’s Q test was used to evaluate heterogeneity. To ascertain potential pleiotropy, we employed both the MR-Egger regression and the MR-PRESSO test. Sensitivity analysis was conducted using the leave-one-out method.

**Results:**

The study found that the class *Bacteroidia* (OR = 0.997, 95% CI: 0.995–0.999, *p* = 0.027), genus *Parabacteroides* (OR = 0.997, 95% CI: 0.994–0.999, *p* = 0.037), order *Bacteroidales* (OR = 0.997, 95% CI: 0.995–0.999, *p* = 0.027), and genus *Veillonella* (OR = 0.998, 95% CI: 0.996–0.999, *p* = 0.046) have protective effects on CM. On the order hand, the genus *Blautia* (OR = 1.003, 95% CI: 1–1.006, *p* = 0.001) and phylum *Cyanobacteria* (OR = 1.002, 95% CI: 1–1.004, *p* = 0.04) are identified as risk factors for CM.

**Conclusion:**

We comprehensively assessed the potential causal relationship between the gut microbiota and CM and identified associations between six gut microbiota and CM. Among these, four gut microbiota were identified as protective factors for CM, while two gut microbiota were identified as risk factors for CM. This study effectively established a causal relationship between the gut microbiota and CM, thereby providing valuable insights into the mechanistic pathways through which the microbiota impacts the progression of CM.

## 1 Introduction

Melanoma, a malignant neoplasm originating from melanocytes, primarily presents in various locations including the skin, choroid of the eye, and the leptomeninges. This disease is distinguished by an unfavorable prognosis, notable invasiveness, and a diminished likelihood of survival. Although cutaneous melanoma (CM) just approximately 5% of all skin malignancies, its tendency to metastasize and spread extensively is responsible for more than 80% of all fatalities associated with skin cancer (Siegel et al., 2022). Lately, there has been a significant rise in the prevalence of melanoma worldwide, especially in Western countries, making it a prominent cancer among people of Caucasian descent. The precise etiological mechanisms underlying melanoma remain incompletely understood. Currently, it is recognized that numerous elements, such as heredity, race, external influences, UV exposure, skin complexion, and various lifestyle factors, may potentially contribute to the development of melanoma. In the past few years, academic discussions have centered around the connection between the gut microbiome and wellbeing of the skin, emphasizing the presence of a gut microbiota-skin relationship. Nevertheless, the observational nature of these studies poses challenges in establishing causality. Therefore, the objective of this research was to assess the causal association between gut microbiome and CM.

In contemporary times, there has been a growing emphasis on the practical significance of the intestinal microbiome within the human organism, acknowledging its function as an additional metabolic organ involved in host metabolic processes (Zhu et al., 2010). Simultaneously, the gut microbiome and its metabolic byproducts assume a pivotal role in maintaining immune system homeostasis within the host organism, as they facilitate immune system maturation, elicit immune responses, and regulate immune cell functionality (Thomas et al., 2017). Studies conducted recently have uncovered a possible connection between the gut microbiota and the probability of cancer development (He et al., 2022; Wei et al., 2022; Yinhang et al., 2022). The authors Zitvogel et al. (2017) carried out research which revealed a correlation between particular elements and amounts of the intestinal microbiota and the development of nine significant types of cancer, including oral, lung, head and neck squamous cell carcinoma, breast, and pancreatic cancer. Notably, changes in gut microbiota have been discovered to be linked with the adverse reactions of PD-1/PD-L1 inhibitors (Routy et al., 2018). Matson et al. (2018) conducted a metagenomic examination on stool samples collected from individuals with melanoma who were receiving ICI therapy, which unveiled *Bifidobacterium longum*, *Enterococcus faecium*, and *Collinsella aerofaciens* as potential microbes that enhance the efficacy of PD-L1 inhibitors. Various research studies have shown the possible impact of different organisms on melanoma, having both positive and negative effects. Nevertheless, because of the complex characteristics and constantly evolving dynamics of the gut microbiota, it is not feasible to solely depend on these discoveries to accurately predict results. Therefore, it is imperative to adopt a new approach for examining the association between the gut microbiota and melanoma.

Mendelian randomization (MR), an analytical method, provides a way to infer likely cause-and-effect connections using observed correlations. The utilization of genetic variations as instrumental variables (IVs) enables the evaluation of the causal influence of exposure on outcomes, it is possible to reduce bias caused by confounding factors or reverse causation (Emdin et al., 2017). Presently, the MR method has garnered significant acclaim in evaluating the causal link between the intestinal microbiome and various ailments (Zhuang et al., 2020; Ni et al., 2021, 2022).

This study aims to investigate the possible causal link between the gut microbiota and CM by utilizing the two-sample MR approach.

## 2 Materials and methods

### 2.1 Design of the study

**FIGURE 1:**
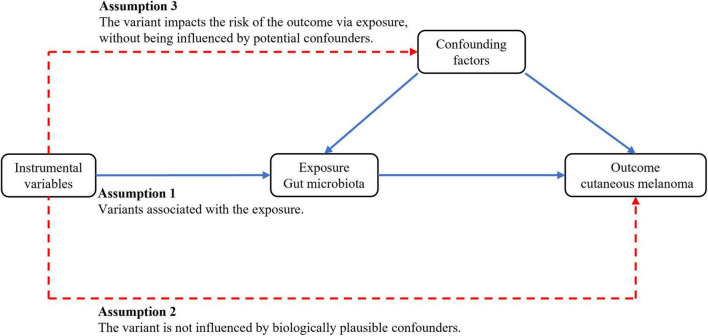
The overview of design.

This study employed publicly available summary statistics from genome-wide association studies (GWAS) for two-sample MR analysis to assess the causal relationship between the gut microbiota and CM. Subsequently, heterogeneity tests, gene pleiotropy tests, and other quality control measures were conducted to validate the reliability of the causal relationship. MR analysis relies on three assumptions: (1) IVs are closely associated with the gut microbiota; (2) IVs are independent of confounding factors influencing CM; (3) IVs solely affect CM through their influence on gut microbiota abundance. The study design is visually depicted in Figure 1.

### 2.2 Sources of data

The gut microbiome GWAS data were obtained through genome-wide statistical analysis conducted on 18,340 individuals of European ethnicity from 11 countries (24 cohorts) as part of the MiBioGen consortium (Kurilshikov et al., 2021). Within the GWAS, there were 211 distinct taxonomic groups and 122,110 variant loci, which covered 9 phyla, 16 classes, 20 orders, 36 families, and 131 genera.

We acquired CM GWAS data (ieu-b-4969) from the ieu-b datasets concerning the outcome data (Bell, 2020). Based on the diagnostic code provided by the International Classification of Diseases-10 (ICD-10), the patient’s condition was verified as CM. The dataset encompasses a total of 375,767 samples and 372,016 controls pertaining to CM, encompassing 11,396,019 single nucleotide polymorphisms (SNPs) that have undergone meticulous scrutiny, including quality control measures such as accurate gene typing and evaluation for Hardy-Weinberg equilibrium. It is important to note that all individuals included in this study were of European descent.

### 2.3 Selection of instrumental variables

In line with modern MR investigations of the gut microbiome, we utilized a threshold of genome-wide significance (*p* < 1 × 10^–5^) to specifically detect SNPs linked to individual gut microbiota (Cao et al., 2023). The specific screening steps were as follows: (1) Utilizing the TwoSampleMR R package to extract relevant SNPs from the summary data of gut microbiota GWAS; (2) To guarantee statistical independence, linkage disequilibrium (LD) analysis was conducted using data from the European division of the 1,000 Genomes Project, with an *R*^2^ value less than 0.001 and a clumping distance of 10,000 kb; (3) To assess the existence of weak instrumental bias, the *F*-statistic was computed for instrumental variables using the formula:


$$F=R^{2}\times \frac{n-k-1}{k\times \left(1-R^{2}\right)}$$

N denotes the sample size, and k indicates the number of IVs, reflecting the strength of the relationship between IVs and the exposure. An *F*-statistic greater than 10 was deemed as proof that there was no bias resulting from weak instrumental variables.

### 2.4 MR analysis

To investigate the causal relationship between CM outcomes and microbiome features, a two-sample MR analysis was performed, integrating data from both host-CM and microbiome-host GWAS. Five widely employed MR methods, namely inverse variance weighted (IVW) test, MR-Egger regression, Weighted Median Estimator, Weighted Mode, and Simple Mode, were utilized (Burgess et al., 2013; Bowden et al., 2015). The IVW method provides unbiased estimates, as long as all genetic variants are valid instruments. MR-Egger analysis is similar to IVW analysis, but the intercept does not have to pass through the origin. The weighted median estimator is used to combine data on multiple genetic variants into a single causal estimate. In addition, we used simple median and weighted median consensus methods, which provide unbiased causal inferences if most genetic instruments are valid. Due to the superior testing efficiency of the IVW method compared to the other four MR methods, we used the IVW method as the principal MR analytical approach (Bowden et al., 2016).

### 2.5 Analysis of horizontal pleiotropy and heterogeneity

To further test the stability and reliability of the results, this study conducted quality control on MR results with FDR-corrected *p*-values less than 0.05. Using the leave-one-out sensitivity analysis approach, each SNP is then left out, and the impact of that SNP is calculated. Heterogeneity testing utilized Cochran’s Q test to assess SNP heterogeneity, evaluating the potential bias in causal effect estimates due to measurement error caused by different analysis platforms, experimental conditions, study populations, etc. Horizontal pleiotropy detection utilized the intercept term of MR-Egger regression to assess whether IVs affect outcomes through pathways other than the exposure.

### 2.6 Data processing

The R software (R.4.2.3) packages TwoSampleMR and MR-PRESSO were used to perform all the mentioned analyses, which included sensitivity analysis and MR analyses.

## 3 Results

### 3.1 SNPs selection

The analysis included a total of 211 characteristics, covering 131 genera, 35 families, 20 orders, 16 classes, and 9 phyla. After implementing quality checks, the quantity of SNPs linked to each bacterial taxonomic category ranged from 3 to 24 (Supplementary Table 1).

### 3.2 MR analyses

When analyzing MR, a comprehensive assessment uncovers statistically significant connections between the risk of CM and six bacterial characteristics, covering various taxonomic levels like phylum, class, order, and genus. A grand total of 70 SNPs were kept in the ultimate selection. The distribution of these genetic variations is as follows: 15 SNPs from the class *Bacteroidia*, 15 SNPs from the order *Bacteroidales*, 9 SNPs from the genus *Parabacteroides*, 9 SNPs from the genus *Veillonella*, 13 SNPs from the genus *Blautia*, and 9 SNPs from the phylum *Cyanobacteria*. It is important to mention that all of these SNPs had *F*-statistics exceeding 10 (Supplementary Table 2).

The results presented in Table 1 demonstrate that the IVW analysis revealed significant associations between the class *Bacteroidia*, genus *Blautia*, genus *Parabacteroides*, phylum *Cyanobacteria*, order *Bacteroidales*, genus *Veillonella*, and the occurrence of CM. The class *Bacteroidia* (OR = 0.997, 95% CI: 0.995–0.999, *p* = 0.027), genus *Parabacteroides* (OR = 0.997, 95% CI: 0.994–0.999, *p* = 0.037), order *Bacteroidales* (OR = 0.997, 95% CI: 0.995–0.999, *p* = 0.027), genus *Veillonella* (OR = 0.998, 95% CI: 0.996–0.999, *p* = 0.046) had protective effects on CM, and the genus *Blautia* (OR = 1.003, 95% CI: 1–1.006, *p* = 0.001), phylum *Cyanobacteria* (OR = 1.002, 95% CI: 1–1.004, *p* = 0.04) were risk factors for CM.

**TABLE 1:** MR results of causal links between gut microbiome and cutaneous melanoma risk.

Group	Gut microbiota	MR method	No. SNP	OR (95% CI)	*p*-value
Class	*Bacteroidia*	Inverse variance weighted	15	0.997 (0.995, 0.999)	0.027
		MR Egger	15	1.001 (0.995, 1.006)	0.833
		Weighted median	15	0.997 (0.994, 1.001)	0.097
		Simple mode	15	0.996 (0.99, 1.002)	0.179
		Weighted mode	15	0.997 (0.993, 1.001)	0.211
Genus	*Blautia*	Inverse variance weighted	13	1.003 (1, 1.006)	0.033
		MR Egger	13	1.003 (0.997, 1.01)	0.312
		Weighted median	13	1.002 (0.998, 1.005)	0.357
		Simple mode	13	1.001 (0.994, 1.007)	0.882
		Weighted mode	13	0.999 (0.994, 1.006)	0.934
Genus	*Parabacteroides*	Inverse variance weighted	9	0.997 (0.994, 0.999)	0.037
		MR Egger	9	0.994 (0.987, 1.002)	0.162
		Weighted median	9	0.996 (0.993, 1)	0.050
		Simple mode	9	0.996 (0.99, 1.002)	0.187
		Weighted mode	9	0.995 (0.99, 1.001)	0.115
Phylum	*Cyanobacteria*	Inverse variance weighted	9	1.002 (1, 1.004)	0.044
		MR Egger	9	1.003 (0.998, 1.009)	0.299
		Weighted median	9	1.002 (1, 1.005)	0.040
		Simple mode	9	1.003 (0.999, 1.006)	0.181
		Weighted mode	9	1.003 (0.999, 1.006)	0.164
Order	*Bacteroidales*	Inverse variance weighted	15	0.997 (0.995, 0.999)	0.027
		MR Egger	15	1.001 (0.995, 1.006)	0.833
		Weighted median	15	0.997 (0.994, 1.001)	0.101
		Simple mode	15	0.996 (0.99, 1.002)	0.195
		Weighted mode	15	0.997 (0.993, 1.001)	0.204
Genus	*Veillonella*	Inverse variance weighted	9	0.998 (0.996, 0.999)	0.046
		MR Egger	9	1.005 (0.994, 1.016)	0.408
		Weighted median	9	0.998 (0.995, 1.001)	0.100
		Simple mode	9	0.997 (0.992, 1.001)	0.209
		Weighted mode	9	0.997 (0.992, 1.002)	0.257

**FIGURE 2:**
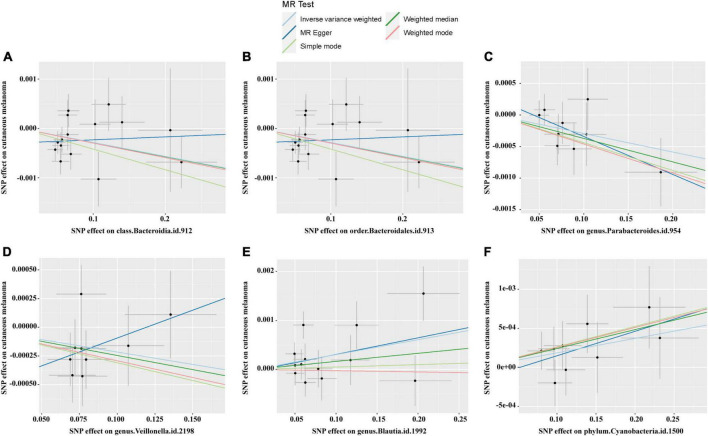
Scatter plots for the causal links between gut microbiota and CM. **(A)** Class *Bacteroidia*; **(B)** order *Bacteroidales*; **(C)** genus *Parabacteroides*; **(D)** genus *Veillonella*; **(E)** genus *Blautia*; **(F)** phylum *Cyanobacteria*.

The causal relationship scatterplot between the gut microbiome and CM is illustrated in Figure 2. The scatterplot illustrates that *Bacteroidia*, *Blautia*, *Parabacteroides*, and *Cyanobacteria* may have a protective effect on CM, while *Bacteroidales* and *Veillonella* may be positively associated with the risk of CM. The IVW, MR-Egger, Weighted median, Weighted mode and Simple mode are MR analysis methods, which are described in the scatterplot. The upward trend of the segments in the scatterplot indicates that this genus of bacteria may be associated with an increased risk of CM, while the downward trend of the segment indicates that this genus of bacteria may be a protective factor for CM.

### 3.3 Sensitivity analyses

The Cochran’s Q statistic was employed to assess heterogeneity. The *p*-values of IVW and MR Egger for *Bacteroidia*, *Blautia*, *Parabacteroides*, *Cyanobacteria*, *Bacteroidales*, and *Veillonella* are all greater than 0.05, indicating the absence of heterogeneity (Supplementary Table 3).

The horizontal pleiotropy between SNPs and outcomes was assessed through MR-Egger regression. The MR-Egger regression intercept *p*-values for *Bacteroidia* (intercept *p* = 0.196), *Blautia* (intercept *p* = 0.904), *Parabacteroides* (intercept *p* = 0.418), *Cyanobacteria* (intercept *p* = 0.642), *Bacteroidales* (intercept *p* = 0.196), and *Veillonella* (intercept *p* = 0.236) were all greater than 0.05, indicating no evidence of horizontal pleiotropy (Supplementary Table 4).

**FIGURE 3:**
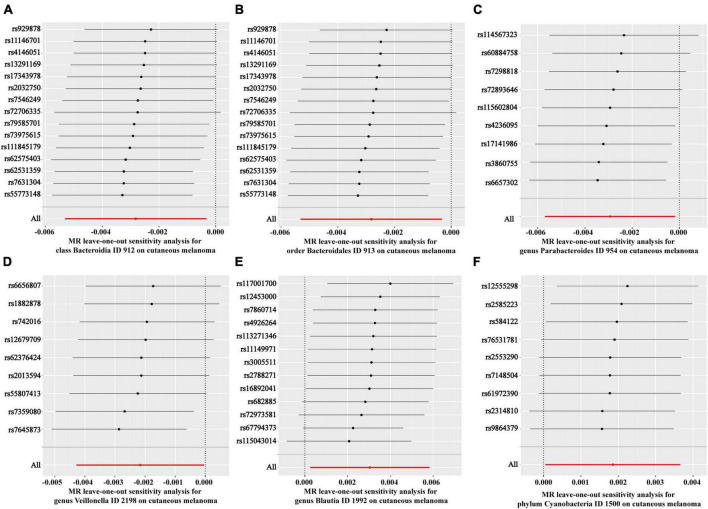
Forest plots of leave-one-out analyses for causal SNP effect of gut microbiota on CM. **(A)** Class *Bacteroidia*; **(B)** order *Bacteroidales*; **(C)** genus *Parabacteroides*; **(D)** genus *Veillonella*; **(E)** genus *Blautia*; **(F)** phylum *Cyanobacteria*.

Subsequently, the leave-one-out method was employed to examine the potential influence of individual SNPs on the estimation of causal relationships. This approach involved systematically removing each SNP to determine if any single SNP exerted a dominant effect on the overall assessment. Figure 3 depicts the sequential exclusion of 15 SNPs associated with *Bacteroidia* (Figure 3A), 15 SNPs associated with *Blautia* (Figure 3B), 9 SNPs associated with *Parabacteroides* (Figure 3C), 9 SNPs associated with *Cyanobacteria* (Figure 3D), 13 SNPs associated with *Bacteroidales* (Figure 3E), and 9 SNPs associated with *Veillonella* (Figure 3F), demonstrating that the exclusion of individual SNPs did not alter the combined effect of the remaining SNPs. These findings suggest a causal relationship between increased abundance of *Bacteroidia*, *Blautia*, *Parabacteroides*, *Cyanobacteria*, *Bacteroidales*, and *Veillonella* and CM.

## 4 Discussion

The implementation of a two-sample MR approach in this study is groundbreaking as it explores the potential causal connection between the gut microbiome and CM. Rigorous quality assurance procedures were utilized for SNPs, resulting in the discovery of a causal association between the susceptibility to CM and six distinct gut microbiome taxa, specifically class *Bacteroidia*, genus *Parabacteroides*, order *Bacteroidales*, genus *Veillonella*, genus *Blautia*, and phylum *Cyanobacteria*.

The class *Bacteroides*, order *Bacteroides*, and genus *Parabacteroides* are all taxonomic groups within the phylum *Bacteroides*. *Bacteroidetes* and *Firmicutes* are the predominant bacterial groups found in the human intestinal tract, with *Bacteroidetes* being more prevalent (Brinkmann et al., 2022). *Bacteroides* have a vital function in multiple important metabolic activities, including glucose processing, utilization of nitrogen-containing substances, and transformation of bile acids and other steroids (Wang et al., 2021; Zafar and Saier, 2021). However, specific strains of *Bacteroides* have been observed to possess both advantageous and detrimental functions, contingent upon their localization within the host. Usually, these varieties provide advantages in the digestive system, but they have the capacity to function as opportunistic pathogens in different parts of the body (Zafar and Saier, 2021). *Bacteroides* have been linked to protecting against liver fibrosis, colitis, immune response to tumors, and cardiovascular disorders (Parker et al., 2020). Nevertheless, the precise involvement of *Bacteroides* in tumorigenesis remains a subject of debate. Although certain research suggests a decrease in the likelihood of developing liver cancer linked to *Bacteroides*, this bacterium has been linked to the development of colon cancer (Parker et al., 2020; Ma et al., 2023). The main concentration of studies regarding the impact of *Bacteroides* on melanoma revolves around its effect on the immune therapy response. The existence of *Bacteroides* in the gastrointestinal tract of individuals with melanoma is linked to an increased incidence of immune-related negative reactions (Andrews et al., 2021). Furthermore, earlier studies have noted a decreased ratio of *Bacteroides* within the gastrointestinal microbiota of melanoma individuals who have displayed a favorable reaction to immunotherapy (Gopalakrishnan et al., 2018; Peters et al., 2019). Although many researches indicate the importance of the gut microbiome in the response to immune therapy, the exact mechanisms involved are still unknown. The examination has uncovered a possible safeguarding impact of the category *Bacteroidia* and the arrangement *Bacteroidales* against CM. Nonetheless, confirming causality requires conducting further comprehensive studies.

In recent times, *Parabacteroides* has been subject to extensive examination as a probiotic. Previous research has demonstrated that *Parabacteroides* exhibits therapeutic potential in the management of various medical conditions, such as obesity, chronic obstructive pulmonary disease, epilepsy, and acute pancreatitis (Wu H. et al., 2022). However, recent research has discovered its potential to induce depressive-like behavior in a mouse model of Crohn’s disease (Wang et al., 2019). The association between the presence of *Parabacteroides* and the progression of cancer has been established, as multiple studies have found a correlation between *Parabacteroides* and the expression of carcinoembryonic antigen (CEA) (Fan et al., 2023). Moreover, a recent MR analysis has presented proof endorsing a causal connection between *Parabacteroides* and breast cancer (Hong et al., 2023). Melanoma research has given little focus to the genus *Parabacteroides*. A recent research study investigated the impact of gut microbiota on the efficacy of combined PD-1 immunotherapy and chemotherapy in patients diagnosed with advanced solid tumors, encompassing melanoma, non-small cell lung cancer, renal cell carcinoma, and hepatocellular carcinoma. The study found a significant increase in the abundance of the genus *Parabacteroides* among patients who showed positive response to the treatment. It can be inferred that the genus *Parabacteroides* might have a significant impact on the effectiveness of immunotherapy and chemotherapy (Wu Z. et al., 2022). The findings of this research suggest that the genus *Parabacteroides* could have a notable impact on the effectiveness of immunotherapy and chemotherapy for treating a specific ailment. Furthermore, it indicates that the genus *Parabacteroides* could potentially act as a safeguard against the progression of CM. Nevertheless, it is crucial to acknowledge the lack of studies exploring the exact mechanisms that link the genus *Parabacteroides* to CM. The study investigating the impact of the genus *Parabacteroides* on the effectiveness of immunotherapy revealed a significant association between the DNA damage response (DDR) pathway, which includes homologous recombination repair, mismatch repair, and non-homologous end joining, and the abundant components of the gut microbiome in individuals who showed a positive response to the treatment. This discovery implies that the microbiome might impact the effectiveness of immunotherapy by means of this specific mechanism, although further research is necessary to completely understand this connection.

*Veillonella*, a coccus that is gram-negative and flourishes in conditions without oxygen, is particularly abundant in the oral. The examination has uncovered a connection between the *Veillonella* category and a reduced vulnerability to CM. Nevertheless, the precise involvement of *Veillonella* in melanoma remains a topic of contention. It is widely acknowledged that a significant feature of immunotherapy is the notable increase in the prevalence of the *Veillonellaceae* family. However, contradictory results have also suggested increased *Veillonella* concentrations in melanoma individuals who demonstrate resistance to immunotherapy (Baruch et al., 2021). Furthermore, *Veillonella* has been linked to a higher vulnerability to lung cancer and gastric cancer, apart from its influence on CM (Yan et al., 2015; Liu D. et al., 2021). The identification of *Veillonella* as a protective factor against CM in this study constitutes a noteworthy revelation. This observation could potentially be attributed to the capacity of *Veillonella* bacteria to impede tumor proliferation through the modulation of nutrient absorption by neoplastic cells. Nevertheless, the existing understanding of *Veillonella* is restricted, demanding additional investigation to uncover the mechanisms through which *Veillonella* functions as a safeguard against melanoma.

Furthermore, this research has uncovered a heightened susceptibility to CM linked to the genus *Blautia* and the phylum *Cyanobacteria*. The detection of *Blautia* has attracted considerable interest due to its ability to reduce inflammatory and metabolic disorders, as well as its effectiveness in combating certain microorganisms (Liu X. et al., 2021). Nevertheless, despite the diverse range of potential probiotic attributes displayed by *Blautia*, our comprehension of this genus remains inadequate. Previous research findings align with this study’s identification of a possible association between *Blautia* and a heightened susceptibility to CM (Peters et al., 2019). Earlier studies have additionally confirmed a link between *Blautia* and a reduced duration of progression-free survival (PFS) in people diagnosed with melanoma (Peters et al., 2019). *Blautia* showed a greater prevalence in hepatocellular carcinoma compared to healthy controls, as observed in a study examining the gut microbiome of 44 patients diagnosed with primary liver cancer and 76 individuals without the disease (Pomyen et al., 2023). However, the specific mechanisms that account for this correlation have yet to be determined.

*Cyanobacteria*, being ancient photosynthetic microorganisms, have the potential to cause various concerns, such as detrimental effects on human skin. Literature reviews suggest that *Cyanobacteria* could pose a novel risk element for melanoma (Drobac Backović et al., 2020). However, there is presently a shortage of studies investigating the mechanistic impact of *Cyanobacteria* on the progression of melanoma. Prior studies have revealed possible cancer-causing pathways of *Cyanobacteria* in relation to colorectal cancer. According to Jiang et al. (2023) *Cyanobacteria* produce a harmful secondary metabolite called Microcystin-LR (MC-LR), which has been found to facilitate the advancement of colorectal cancer. This occurs through the upregulation of TGF-β1 expression and secretion in M2 macrophages, while simultaneously downregulating CST3 in colorectal cancer cells (Jiang et al., 2023). Nevertheless, it is crucial to acknowledge that additional evidence is required to establish a conclusive link between *Cyanobacteria* and the carcinogenic processes associated with other types of cancer.

This study had several limitations. Firstly, the examination of the gut microbiome solely distinguished at the phylum to genus hierarchy, disregarding more precise taxonomic levels. Secondly, the results are limited to people of European descent and do not include differences in gut microbiomes among various ethnic groups. Lastly, the inquiry failed to incorporate an examination of the intestinal microbiota in connection with alternative types of melanoma, underscoring the necessity for additional investigation in this domain.

The aim of this study was to assess the potential involvement of the intestinal microbiome in CM. However, given the intricate composition of the gut microbiota, it is advisable to exercise caution when interpreting its causal associations. The results of this research could offer valuable knowledge on the detection of metabolites and biomarkers originating from fecal microbiota. This information may assist in the timely identification of CM and the assessment of melanoma’s reaction to immunotherapy.

## Data availability statement

The original contributions presented in this study are included in the article/Supplementary material, further inquiries can be directed to the corresponding author.

## Ethics statement

The studies involving humans were approved by the original genome-wide association studies (GWAS) contains the necessary ethical approval and participant consent. The studies were conducted in accordance with the local legislation and institutional requirements. The participants provided their written informed consent to participate in this study.

## Author contributions

Y-QB: Formal analysis, Methodology, Writing – original draft. YZ: Formal analysis, Investigation, Methodology, Software, Writing – original draft. Z-NL: Conceptualization, Methodology, Project administration, Writing – review & editing.
