# Supplementary material for: Causal associations between gut microbiota and cutaneous melanoma: a Mendelian randomization study
Source: Front Microbiol. 2024 Apr 8;15:1339621. doi: 10.3389/fmicb.2024.1339621 (PMC11033470; doi:10.3389/fmicb.2024.1339621)
Supplement: Supplementary file 1 [file Table_1.DOCX]

***Supplementary Material***

Table S1 Full MR results of causal links between gut microbiome and cutaneous melanoma risk.

| id.exposure | MR method | No. SNP | *p*-val |
| --- | --- | --- | --- |
| class.Alphaproteobacteria.id.2379 | MR Egger | 9 | 0.901 |
| class.Alphaproteobacteria.id.2379 | Weighted median | 9 | 0.340 |
| class.Alphaproteobacteria.id.2379 | Inverse variance weighted | 9 | 0.190 |
| class.Alphaproteobacteria.id.2379 | Simple mode | 9 | 0.451 |
| class.Alphaproteobacteria.id.2379 | Weighted mode | 9 | 0.445 |
| class.Actinobacteria.id.419 | MR Egger | 21 | 0.192 |
| class.Actinobacteria.id.419 | Weighted median | 21 | 0.126 |
| class.Actinobacteria.id.419 | Inverse variance weighted | 21 | 0.126 |
| class.Actinobacteria.id.419 | Simple mode | 21 | 0.156 |
| class.Actinobacteria.id.419 | Weighted mode | 21 | 0.177 |
| class.Bacilli.id.1673 | MR Egger | 20 | 0.991 |
| class.Bacilli.id.1673 | Weighted median | 20 | 0.869 |
| class.Bacilli.id.1673 | Inverse variance weighted | 20 | 0.847 |
| class.Bacilli.id.1673 | Simple mode | 20 | 0.716 |
| class.Bacilli.id.1673 | Weighted mode | 20 | 0.779 |
| class.Clostridia.id.1859 | MR Egger | 16 | 0.509 |
| class.Clostridia.id.1859 | Weighted median | 16 | 0.983 |
| class.Clostridia.id.1859 | Inverse variance weighted | 16 | 0.966 |
| class.Clostridia.id.1859 | Simple mode | 16 | 0.529 |
| class.Clostridia.id.1859 | Weighted mode | 16 | 0.613 |
| class.Betaproteobacteria.id.2867 | MR Egger | 15 | 0.909 |
| class.Betaproteobacteria.id.2867 | Weighted median | 15 | 0.554 |
| class.Betaproteobacteria.id.2867 | Inverse variance weighted | 15 | 0.733 |
| class.Betaproteobacteria.id.2867 | Simple mode | 15 | 0.363 |
| class.Betaproteobacteria.id.2867 | Weighted mode | 15 | 0.410 |
| class.Bacteroidia.id.912 | MR Egger | 15 | 0.833 |
| class.Bacteroidia.id.912 | Weighted median | 15 | 0.097 |
| class.Bacteroidia.id.912 | Inverse variance weighted | 15 | 0.027 |
| class.Bacteroidia.id.912 | Simple mode | 15 | 0.179 |
| class.Bacteroidia.id.912 | Weighted mode | 15 | 0.211 |
| family.FamilyXIII.id.1957 | MR Egger | 10 | 0.541 |
| family.FamilyXIII.id.1957 | Weighted median | 10 | 0.665 |
| family.FamilyXIII.id.1957 | Inverse variance weighted | 10 | 0.624 |
| family.FamilyXIII.id.1957 | Simple mode | 10 | 0.562 |
| family.FamilyXIII.id.1957 | Weighted mode | 10 | 0.628 |
| family.FamilyXI.id.1936 | MR Egger | 10 | 0.785 |
| family.FamilyXI.id.1936 | Weighted median | 10 | 0.486 |
| family.FamilyXI.id.1936 | Inverse variance weighted | 10 | 0.985 |
| family.FamilyXI.id.1936 | Simple mode | 10 | 0.606 |
| family.FamilyXI.id.1936 | Weighted mode | 10 | 0.599 |
| family.Erysipelotrichaceae.id.2149 | MR Egger | 13 | 0.084 |
| family.Erysipelotrichaceae.id.2149 | Weighted median | 13 | 0.610 |
| family.Erysipelotrichaceae.id.2149 | Inverse variance weighted | 13 | 0.664 |
| family.Erysipelotrichaceae.id.2149 | Simple mode | 13 | 0.250 |
| family.Erysipelotrichaceae.id.2149 | Weighted mode | 13 | 0.225 |
| family.Enterobacteriaceae.id.3469 | MR Egger | 10 | 0.475 |
| family.Enterobacteriaceae.id.3469 | Weighted median | 10 | 0.459 |
| family.Enterobacteriaceae.id.3469 | Inverse variance weighted | 10 | 0.230 |
| family.Enterobacteriaceae.id.3469 | Simple mode | 10 | 0.597 |
| family.Enterobacteriaceae.id.3469 | Weighted mode | 10 | 0.617 |
| family.Desulfovibrionaceae.id.3169 | MR Egger | 12 | 0.359 |
| family.Desulfovibrionaceae.id.3169 | Weighted median | 12 | 0.085 |
| family.Desulfovibrionaceae.id.3169 | Inverse variance weighted | 12 | 0.308 |
| family.Desulfovibrionaceae.id.3169 | Simple mode | 12 | 0.208 |
| family.Desulfovibrionaceae.id.3169 | Weighted mode | 12 | 0.145 |
| family.Defluviitaleaceae.id.1924 | MR Egger | 9 | 0.282 |
| family.Defluviitaleaceae.id.1924 | Weighted median | 9 | 0.178 |
| family.Defluviitaleaceae.id.1924 | Inverse variance weighted | 9 | 0.368 |
| family.Defluviitaleaceae.id.1924 | Simple mode | 9 | 0.454 |
| family.Defluviitaleaceae.id.1924 | Weighted mode | 9 | 0.310 |
| family.Coriobacteriaceae.id.811 | MR Egger | 19 | 0.747 |
| family.Coriobacteriaceae.id.811 | Weighted median | 19 | 0.824 |
| family.Coriobacteriaceae.id.811 | Inverse variance weighted | 19 | 0.980 |
| family.Coriobacteriaceae.id.811 | Simple mode | 19 | 0.760 |
| family.Coriobacteriaceae.id.811 | Weighted mode | 19 | 0.974 |
| family.ClostridialesvadinBB60group.id.11286 | MR Egger | 15 | 0.934 |
| family.ClostridialesvadinBB60group.id.11286 | Weighted median | 15 | 0.890 |
| family.ClostridialesvadinBB60group.id.11286 | Inverse variance weighted | 15 | 0.839 |
| family.ClostridialesvadinBB60group.id.11286 | Simple mode | 15 | 0.808 |
| family.ClostridialesvadinBB60group.id.11286 | Weighted mode | 15 | 0.854 |
| family.Clostridiaceae1.id.1869 | MR Egger | 11 | 0.871 |
| family.Clostridiaceae1.id.1869 | Weighted median | 11 | 0.614 |
| family.Clostridiaceae1.id.1869 | Inverse variance weighted | 11 | 0.167 |
| family.Clostridiaceae1.id.1869 | Simple mode | 11 | 0.960 |
| family.Clostridiaceae1.id.1869 | Weighted mode | 11 | 0.840 |
| family.Christensenellaceae.id.1866 | MR Egger | 10 | 0.829 |
| family.Christensenellaceae.id.1866 | Weighted median | 10 | 0.852 |
| family.Christensenellaceae.id.1866 | Inverse variance weighted | 10 | 0.739 |
| family.Christensenellaceae.id.1866 | Simple mode | 10 | 0.806 |
| family.Christensenellaceae.id.1866 | Weighted mode | 10 | 0.834 |
| family.Bifidobacteriaceae.id.433 | MR Egger | 21 | 0.553 |
| family.Bifidobacteriaceae.id.433 | Weighted median | 21 | 0.293 |
| family.Bifidobacteriaceae.id.433 | Inverse variance weighted | 21 | 0.325 |
| family.Bifidobacteriaceae.id.433 | Simple mode | 21 | 0.591 |
| family.Bifidobacteriaceae.id.433 | Weighted mode | 21 | 0.390 |
| family.BacteroidalesS24.7group.id.11173 | MR Egger | 9 | 0.604 |
| family.BacteroidalesS24.7group.id.11173 | Weighted median | 9 | 0.835 |
| family.BacteroidalesS24.7group.id.11173 | Inverse variance weighted | 9 | 0.883 |
| family.BacteroidalesS24.7group.id.11173 | Simple mode | 9 | 0.833 |
| family.BacteroidalesS24.7group.id.11173 | Weighted mode | 9 | 0.819 |
| family.Bacteroidaceae.id.917 | MR Egger | 10 | 0.870 |
| family.Bacteroidaceae.id.917 | Weighted median | 10 | 0.770 |
| family.Bacteroidaceae.id.917 | Inverse variance weighted | 10 | 0.390 |
| family.Bacteroidaceae.id.917 | Simple mode | 10 | 0.902 |
| family.Bacteroidaceae.id.917 | Weighted mode | 10 | 0.805 |
| family.Alcaligenaceae.id.2875 | MR Egger | 18 | 0.192 |
| family.Alcaligenaceae.id.2875 | Weighted median | 18 | 0.348 |
| family.Alcaligenaceae.id.2875 | Inverse variance weighted | 18 | 0.984 |
| family.Alcaligenaceae.id.2875 | Simple mode | 18 | 0.315 |
| family.Alcaligenaceae.id.2875 | Weighted mode | 18 | 0.284 |
| family.Actinomycetaceae.id.421 | MR Egger | 5 | 0.616 |
| family.Actinomycetaceae.id.421 | Weighted median | 5 | 0.461 |
| family.Actinomycetaceae.id.421 | Inverse variance weighted | 5 | 0.235 |
| family.Actinomycetaceae.id.421 | Simple mode | 5 | 0.399 |
| family.Actinomycetaceae.id.421 | Weighted mode | 5 | 0.493 |
| family.Acidaminococcaceae.id.2166 | MR Egger | 7 | 0.384 |
| family.Acidaminococcaceae.id.2166 | Weighted median | 7 | 0.591 |
| family.Acidaminococcaceae.id.2166 | Inverse variance weighted | 7 | 0.587 |
| family.Acidaminococcaceae.id.2166 | Simple mode | 7 | 0.270 |
| family.Acidaminococcaceae.id.2166 | Weighted mode | 7 | 0.282 |
| class.Verrucomicrobiae.id.4029 | MR Egger | 13 | 0.916 |
| class.Verrucomicrobiae.id.4029 | Weighted median | 13 | 0.767 |
| class.Verrucomicrobiae.id.4029 | Inverse variance weighted | 13 | 0.956 |
| class.Verrucomicrobiae.id.4029 | Simple mode | 13 | 0.787 |
| class.Verrucomicrobiae.id.4029 | Weighted mode | 13 | 0.813 |
| class.Negativicutes.id.2164 | MR Egger | 12 | 0.372 |
| class.Negativicutes.id.2164 | Weighted median | 12 | 0.977 |
| class.Negativicutes.id.2164 | Inverse variance weighted | 12 | 0.784 |
| class.Negativicutes.id.2164 | Simple mode | 12 | 0.514 |
| class.Negativicutes.id.2164 | Weighted mode | 12 | 0.663 |
| class.Mollicutes.id.3920 | MR Egger | 12 | 0.340 |
| class.Mollicutes.id.3920 | Weighted median | 12 | 0.907 |
| class.Mollicutes.id.3920 | Inverse variance weighted | 12 | 0.537 |
| class.Mollicutes.id.3920 | Simple mode | 12 | 0.420 |
| class.Mollicutes.id.3920 | Weighted mode | 12 | 0.589 |
| class.Methanobacteria.id.119 | MR Egger | 12 | 0.160 |
| class.Methanobacteria.id.119 | Weighted median | 12 | 0.271 |
| class.Methanobacteria.id.119 | Inverse variance weighted | 12 | 0.552 |
| class.Methanobacteria.id.119 | Simple mode | 12 | 0.501 |
| class.Methanobacteria.id.119 | Weighted mode | 12 | 0.489 |
| class.Melainabacteria.id.1589 | MR Egger | 12 | 0.737 |
| class.Melainabacteria.id.1589 | Weighted median | 12 | 0.921 |
| class.Melainabacteria.id.1589 | Inverse variance weighted | 12 | 0.355 |
| class.Melainabacteria.id.1589 | Simple mode | 12 | 0.603 |
| class.Melainabacteria.id.1589 | Weighted mode | 12 | 0.595 |
| class.Lentisphaeria.id.2250 | MR Egger | 10 | 0.767 |
| class.Lentisphaeria.id.2250 | Weighted median | 10 | 0.165 |
| class.Lentisphaeria.id.2250 | Inverse variance weighted | 10 | 0.491 |
| class.Lentisphaeria.id.2250 | Simple mode | 10 | 0.141 |
| class.Lentisphaeria.id.2250 | Weighted mode | 10 | 0.175 |
| class.Gammaproteobacteria.id.3303 | MR Egger | 8 | 0.281 |
| class.Gammaproteobacteria.id.3303 | Weighted median | 8 | 0.667 |
| class.Gammaproteobacteria.id.3303 | Inverse variance weighted | 8 | 0.472 |
| class.Gammaproteobacteria.id.3303 | Simple mode | 8 | 0.521 |
| class.Gammaproteobacteria.id.3303 | Weighted mode | 8 | 0.504 |
| class.Erysipelotrichia.id.2147 | MR Egger | 13 | 0.084 |
| class.Erysipelotrichia.id.2147 | Weighted median | 13 | 0.613 |
| class.Erysipelotrichia.id.2147 | Inverse variance weighted | 13 | 0.664 |
| class.Erysipelotrichia.id.2147 | Simple mode | 13 | 0.234 |
| class.Erysipelotrichia.id.2147 | Weighted mode | 13 | 0.225 |
| class.Deltaproteobacteria.id.3087 | MR Egger | 14 | 0.261 |
| class.Deltaproteobacteria.id.3087 | Weighted median | 14 | 0.362 |
| class.Deltaproteobacteria.id.3087 | Inverse variance weighted | 14 | 0.392 |
| class.Deltaproteobacteria.id.3087 | Simple mode | 14 | 0.276 |
| class.Deltaproteobacteria.id.3087 | Weighted mode | 14 | 0.213 |
| class.Coriobacteriia.id.809 | MR Egger | 19 | 0.747 |
| class.Coriobacteriia.id.809 | Weighted median | 19 | 0.817 |
| class.Coriobacteriia.id.809 | Inverse variance weighted | 19 | 0.980 |
| class.Coriobacteriia.id.809 | Simple mode | 19 | 0.759 |
| class.Coriobacteriia.id.809 | Weighted mode | 19 | 0.972 |
| genus..Ruminococcusgnavusgroup.id.14376 | MR Egger | 12 | 0.466 |
| genus..Ruminococcusgnavusgroup.id.14376 | Weighted median | 12 | 0.341 |
| genus..Ruminococcusgnavusgroup.id.14376 | Inverse variance weighted | 12 | 0.300 |
| genus..Ruminococcusgnavusgroup.id.14376 | Simple mode | 12 | 0.637 |
| genus..Ruminococcusgnavusgroup.id.14376 | Weighted mode | 12 | 0.652 |
| genus..Ruminococcusgauvreauiigroup.id.11342 | MR Egger | 12 | 0.707 |
| genus..Ruminococcusgauvreauiigroup.id.11342 | Weighted median | 12 | 0.127 |
| genus..Ruminococcusgauvreauiigroup.id.11342 | Inverse variance weighted | 12 | 0.472 |
| genus..Ruminococcusgauvreauiigroup.id.11342 | Simple mode | 12 | 0.171 |
| genus..Ruminococcusgauvreauiigroup.id.11342 | Weighted mode | 12 | 0.198 |
| genus..Eubacteriumxylanophilumgroup.id.14375 | MR Egger | 10 | 0.912 |
| genus..Eubacteriumxylanophilumgroup.id.14375 | Weighted median | 10 | 0.149 |
| genus..Eubacteriumxylanophilumgroup.id.14375 | Inverse variance weighted | 10 | 0.506 |
| genus..Eubacteriumxylanophilumgroup.id.14375 | Simple mode | 10 | 0.296 |
| genus..Eubacteriumxylanophilumgroup.id.14375 | Weighted mode | 10 | 0.311 |
| genus..Eubacteriumventriosumgroup.id.11341 | MR Egger | 13 | 0.706 |
| genus..Eubacteriumventriosumgroup.id.11341 | Weighted median | 13 | 0.177 |
| genus..Eubacteriumventriosumgroup.id.11341 | Inverse variance weighted | 13 | 0.400 |
| genus..Eubacteriumventriosumgroup.id.11341 | Simple mode | 13 | 0.346 |
| genus..Eubacteriumventriosumgroup.id.11341 | Weighted mode | 13 | 0.317 |
| genus..Eubacteriumruminantiumgroup.id.11340 | MR Egger | 16 | 0.424 |
| genus..Eubacteriumruminantiumgroup.id.11340 | Weighted median | 16 | 0.632 |
| genus..Eubacteriumruminantiumgroup.id.11340 | Inverse variance weighted | 16 | 0.681 |
| genus..Eubacteriumruminantiumgroup.id.11340 | Simple mode | 16 | 0.303 |
| genus..Eubacteriumruminantiumgroup.id.11340 | Weighted mode | 16 | 0.349 |
| genus..Eubacteriumrectalegroup.id.14374 | MR Egger | 13 | 0.456 |
| genus..Eubacteriumrectalegroup.id.14374 | Weighted median | 13 | 0.193 |
| genus..Eubacteriumrectalegroup.id.14374 | Inverse variance weighted | 13 | 0.121 |
| genus..Eubacteriumrectalegroup.id.14374 | Simple mode | 13 | 0.259 |
| genus..Eubacteriumrectalegroup.id.14374 | Weighted mode | 13 | 0.652 |
| genus..Eubacteriumoxidoreducensgroup.id.11339 | MR Egger | 5 | 0.086 |
| genus..Eubacteriumoxidoreducensgroup.id.11339 | Weighted median | 5 | 0.940 |
| genus..Eubacteriumoxidoreducensgroup.id.11339 | Inverse variance weighted | 5 | 0.183 |
| genus..Eubacteriumoxidoreducensgroup.id.11339 | Simple mode | 5 | 0.861 |
| genus..Eubacteriumoxidoreducensgroup.id.11339 | Weighted mode | 5 | 0.856 |
| genus..Eubacteriumnodatumgroup.id.11297 | MR Egger | 10 | 0.025 |
| genus..Eubacteriumnodatumgroup.id.11297 | Weighted median | 10 | 0.514 |
| genus..Eubacteriumnodatumgroup.id.11297 | Inverse variance weighted | 10 | 0.850 |
| genus..Eubacteriumnodatumgroup.id.11297 | Simple mode | 10 | 0.376 |
| genus..Eubacteriumnodatumgroup.id.11297 | Weighted mode | 10 | 0.655 |
| genus..Eubacteriumhalliigroup.id.11338 | MR Egger | 15 | 0.259 |
| genus..Eubacteriumhalliigroup.id.11338 | Weighted median | 15 | 0.156 |
| genus..Eubacteriumhalliigroup.id.11338 | Inverse variance weighted | 15 | 0.324 |
| genus..Eubacteriumhalliigroup.id.11338 | Simple mode | 15 | 0.155 |
| genus..Eubacteriumhalliigroup.id.11338 | Weighted mode | 15 | 0.124 |
| genus..Eubacteriumfissicatenagroup.id.14373 | MR Egger | 9 | 0.341 |
| genus..Eubacteriumfissicatenagroup.id.14373 | Weighted median | 9 | 0.812 |
| genus..Eubacteriumfissicatenagroup.id.14373 | Inverse variance weighted | 9 | 0.328 |
| genus..Eubacteriumfissicatenagroup.id.14373 | Simple mode | 9 | 0.876 |
| genus..Eubacteriumfissicatenagroup.id.14373 | Weighted mode | 9 | 0.877 |
| genus..Eubacteriumeligensgroup.id.14372 | MR Egger | 10 | 0.358 |
| genus..Eubacteriumeligensgroup.id.14372 | Weighted median | 10 | 0.518 |
| genus..Eubacteriumeligensgroup.id.14372 | Inverse variance weighted | 10 | 0.557 |
| genus..Eubacteriumeligensgroup.id.14372 | Simple mode | 10 | 0.536 |
| genus..Eubacteriumeligensgroup.id.14372 | Weighted mode | 10 | 0.429 |
| genus..Eubacteriumcoprostanoligenesgroup.id.11375 | MR Egger | 14 | 0.604 |
| genus..Eubacteriumcoprostanoligenesgroup.id.11375 | Weighted median | 14 | 0.572 |
| genus..Eubacteriumcoprostanoligenesgroup.id.11375 | Inverse variance weighted | 14 | 0.945 |
| genus..Eubacteriumcoprostanoligenesgroup.id.11375 | Simple mode | 14 | 0.314 |
| genus..Eubacteriumcoprostanoligenesgroup.id.11375 | Weighted mode | 14 | 0.622 |
| genus..Eubacteriumbrachygroup.id.11296 | MR Egger | 10 | 0.927 |
| genus..Eubacteriumbrachygroup.id.11296 | Weighted median | 10 | 0.753 |
| genus..Eubacteriumbrachygroup.id.11296 | Inverse variance weighted | 10 | 0.526 |
| genus..Eubacteriumbrachygroup.id.11296 | Simple mode | 10 | 0.807 |
| genus..Eubacteriumbrachygroup.id.11296 | Weighted mode | 10 | 0.766 |
| genus..Clostridiuminnocuumgroup.id.14397 | MR Egger | 10 | 0.853 |
| genus..Clostridiuminnocuumgroup.id.14397 | Weighted median | 10 | 0.347 |
| genus..Clostridiuminnocuumgroup.id.14397 | Inverse variance weighted | 10 | 0.146 |
| genus..Clostridiuminnocuumgroup.id.14397 | Simple mode | 10 | 0.605 |
| genus..Clostridiuminnocuumgroup.id.14397 | Weighted mode | 10 | 0.769 |
| family.Victivallaceae.id.2255 | MR Egger | 14 | 0.239 |
| family.Victivallaceae.id.2255 | Weighted median | 14 | 0.618 |
| family.Victivallaceae.id.2255 | Inverse variance weighted | 14 | 0.879 |
| family.Victivallaceae.id.2255 | Simple mode | 14 | 0.682 |
| family.Victivallaceae.id.2255 | Weighted mode | 14 | 0.602 |
| family.Verrucomicrobiaceae.id.4036 | MR Egger | 13 | 0.918 |
| family.Verrucomicrobiaceae.id.4036 | Weighted median | 13 | 0.769 |
| family.Verrucomicrobiaceae.id.4036 | Inverse variance weighted | 13 | 0.956 |
| family.Verrucomicrobiaceae.id.4036 | Simple mode | 13 | 0.771 |
| family.Verrucomicrobiaceae.id.4036 | Weighted mode | 13 | 0.813 |
| family.Veillonellaceae.id.2172 | MR Egger | 19 | 0.056 |
| family.Veillonellaceae.id.2172 | Weighted median | 19 | 0.387 |
| family.Veillonellaceae.id.2172 | Inverse variance weighted | 19 | 0.944 |
| family.Veillonellaceae.id.2172 | Simple mode | 19 | 0.944 |
| family.Veillonellaceae.id.2172 | Weighted mode | 19 | 0.450 |
| family.unknownfamily.id.1000006161 | MR Egger | 15 | 0.458 |
| family.unknownfamily.id.1000006161 | Weighted median | 15 | 0.096 |
| family.unknownfamily.id.1000006161 | Inverse variance weighted | 15 | 0.072 |
| family.unknownfamily.id.1000006161 | Simple mode | 15 | 0.245 |
| family.unknownfamily.id.1000006161 | Weighted mode | 15 | 0.229 |
| family.unknownfamily.id.1000005471 | MR Egger | 14 | 0.614 |
| family.unknownfamily.id.1000005471 | Weighted median | 14 | 0.877 |
| family.unknownfamily.id.1000005471 | Inverse variance weighted | 14 | 0.245 |
| family.unknownfamily.id.1000005471 | Simple mode | 14 | 0.560 |
| family.unknownfamily.id.1000005471 | Weighted mode | 14 | 0.462 |
| family.unknownfamily.id.1000001214 | MR Egger | 11 | 0.726 |
| family.unknownfamily.id.1000001214 | Weighted median | 11 | 0.580 |
| family.unknownfamily.id.1000001214 | Inverse variance weighted | 11 | 0.139 |
| family.unknownfamily.id.1000001214 | Simple mode | 11 | 0.755 |
| family.unknownfamily.id.1000001214 | Weighted mode | 11 | 0.677 |
| family.Streptococcaceae.id.1850 | MR Egger | 15 | 0.166 |
| family.Streptococcaceae.id.1850 | Weighted median | 15 | 0.118 |
| family.Streptococcaceae.id.1850 | Inverse variance weighted | 15 | 0.188 |
| family.Streptococcaceae.id.1850 | Simple mode | 15 | 0.165 |
| family.Streptococcaceae.id.1850 | Weighted mode | 15 | 0.158 |
| family.Ruminococcaceae.id.2050 | MR Egger | 11 | 0.824 |
| family.Ruminococcaceae.id.2050 | Weighted median | 11 | 0.363 |
| family.Ruminococcaceae.id.2050 | Inverse variance weighted | 11 | 0.610 |
| family.Ruminococcaceae.id.2050 | Simple mode | 11 | 0.716 |
| family.Ruminococcaceae.id.2050 | Weighted mode | 11 | 0.497 |
| family.Rikenellaceae.id.967 | MR Egger | 22 | 0.591 |
| family.Rikenellaceae.id.967 | Weighted median | 22 | 0.854 |
| family.Rikenellaceae.id.967 | Inverse variance weighted | 22 | 0.958 |
| family.Rikenellaceae.id.967 | Simple mode | 22 | 0.646 |
| family.Rikenellaceae.id.967 | Weighted mode | 22 | 0.791 |
| family.Rhodospirillaceae.id.2717 | MR Egger | 17 | 0.058 |
| family.Rhodospirillaceae.id.2717 | Weighted median | 17 | 0.620 |
| family.Rhodospirillaceae.id.2717 | Inverse variance weighted | 17 | 0.834 |
| family.Rhodospirillaceae.id.2717 | Simple mode | 17 | 0.417 |
| family.Rhodospirillaceae.id.2717 | Weighted mode | 17 | 0.437 |
| family.Prevotellaceae.id.960 | MR Egger | 17 | 0.763 |
| family.Prevotellaceae.id.960 | Weighted median | 17 | 0.739 |
| family.Prevotellaceae.id.960 | Inverse variance weighted | 17 | 0.865 |
| family.Prevotellaceae.id.960 | Simple mode | 17 | 0.937 |
| family.Prevotellaceae.id.960 | Weighted mode | 17 | 0.852 |
| family.Porphyromonadaceae.id.943 | MR Egger | 11 | 0.524 |
| family.Porphyromonadaceae.id.943 | Weighted median | 11 | 0.350 |
| family.Porphyromonadaceae.id.943 | Inverse variance weighted | 11 | 0.149 |
| family.Porphyromonadaceae.id.943 | Simple mode | 11 | 0.803 |
| family.Porphyromonadaceae.id.943 | Weighted mode | 11 | 0.677 |
| family.Peptostreptococcaceae.id.2042 | MR Egger | 14 | 0.461 |
| family.Peptostreptococcaceae.id.2042 | Weighted median | 14 | 0.815 |
| family.Peptostreptococcaceae.id.2042 | Inverse variance weighted | 14 | 0.836 |
| family.Peptostreptococcaceae.id.2042 | Simple mode | 14 | 0.473 |
| family.Peptostreptococcaceae.id.2042 | Weighted mode | 14 | 0.345 |
| family.Peptococcaceae.id.2024 | MR Egger | 9 | 0.443 |
| family.Peptococcaceae.id.2024 | Weighted median | 9 | 0.147 |
| family.Peptococcaceae.id.2024 | Inverse variance weighted | 9 | 0.124 |
| family.Peptococcaceae.id.2024 | Simple mode | 9 | 0.905 |
| family.Peptococcaceae.id.2024 | Weighted mode | 9 | 0.255 |
| family.Pasteurellaceae.id.3689 | MR Egger | 17 | 0.451 |
| family.Pasteurellaceae.id.3689 | Weighted median | 17 | 0.814 |
| family.Pasteurellaceae.id.3689 | Inverse variance weighted | 17 | 0.605 |
| family.Pasteurellaceae.id.3689 | Simple mode | 17 | 0.879 |
| family.Pasteurellaceae.id.3689 | Weighted mode | 17 | 0.808 |
| family.Oxalobacteraceae.id.2966 | MR Egger | 15 | 0.800 |
| family.Oxalobacteraceae.id.2966 | Weighted median | 15 | 0.446 |
| family.Oxalobacteraceae.id.2966 | Inverse variance weighted | 15 | 0.457 |
| family.Oxalobacteraceae.id.2966 | Simple mode | 15 | 0.471 |
| family.Oxalobacteraceae.id.2966 | Weighted mode | 15 | 0.496 |
| family.Methanobacteriaceae.id.121 | MR Egger | 12 | 0.160 |
| family.Methanobacteriaceae.id.121 | Weighted median | 12 | 0.260 |
| family.Methanobacteriaceae.id.121 | Inverse variance weighted | 12 | 0.552 |
| family.Methanobacteriaceae.id.121 | Simple mode | 12 | 0.515 |
| family.Methanobacteriaceae.id.121 | Weighted mode | 12 | 0.485 |
| family.Lactobacillaceae.id.1836 | MR Egger | 11 | 0.431 |
| family.Lactobacillaceae.id.1836 | Weighted median | 11 | 0.516 |
| family.Lactobacillaceae.id.1836 | Inverse variance weighted | 11 | 0.349 |
| family.Lactobacillaceae.id.1836 | Simple mode | 11 | 0.826 |
| family.Lactobacillaceae.id.1836 | Weighted mode | 11 | 0.911 |
| family.Lachnospiraceae.id.1987 | MR Egger | 14 | 0.883 |
| family.Lachnospiraceae.id.1987 | Weighted median | 14 | 0.851 |
| family.Lachnospiraceae.id.1987 | Inverse variance weighted | 14 | 0.812 |
| family.Lachnospiraceae.id.1987 | Simple mode | 14 | 0.763 |
| family.Lachnospiraceae.id.1987 | Weighted mode | 14 | 0.798 |
| genus.Eisenbergiella.id.11304 | MR Egger | 11 | 0.024 |
| genus.Eisenbergiella.id.11304 | Weighted median | 11 | 0.644 |
| genus.Eisenbergiella.id.11304 | Inverse variance weighted | 11 | 0.858 |
| genus.Eisenbergiella.id.11304 | Simple mode | 11 | 0.877 |
| genus.Eisenbergiella.id.11304 | Weighted mode | 11 | 0.892 |
| genus.Eggerthella.id.819 | MR Egger | 9 | 0.679 |
| genus.Eggerthella.id.819 | Weighted median | 9 | 0.796 |
| genus.Eggerthella.id.819 | Inverse variance weighted | 9 | 0.873 |
| genus.Eggerthella.id.819 | Simple mode | 9 | 0.879 |
| genus.Eggerthella.id.819 | Weighted mode | 9 | 0.888 |
| genus.Dorea.id.1997 | MR Egger | 12 | 0.036 |
| genus.Dorea.id.1997 | Weighted median | 12 | 0.840 |
| genus.Dorea.id.1997 | Inverse variance weighted | 12 | 0.913 |
| genus.Dorea.id.1997 | Simple mode | 12 | 0.958 |
| genus.Dorea.id.1997 | Weighted mode | 12 | 0.889 |
| genus.Dialister.id.2183 | MR Egger | 12 | 0.277 |
| genus.Dialister.id.2183 | Weighted median | 12 | 0.194 |
| genus.Dialister.id.2183 | Inverse variance weighted | 12 | 0.281 |
| genus.Dialister.id.2183 | Simple mode | 12 | 0.190 |
| genus.Dialister.id.2183 | Weighted mode | 12 | 0.162 |
| genus.Desulfovibrio.id.3173 | MR Egger | 11 | 0.159 |
| genus.Desulfovibrio.id.3173 | Weighted median | 11 | 0.480 |
| genus.Desulfovibrio.id.3173 | Inverse variance weighted | 11 | 0.935 |
| genus.Desulfovibrio.id.3173 | Simple mode | 11 | 0.375 |
| genus.Desulfovibrio.id.3173 | Weighted mode | 11 | 0.389 |
| genus.DefluviitaleaceaeUCG011.id.11287 | MR Egger | 8 | 0.499 |
| genus.DefluviitaleaceaeUCG011.id.11287 | Weighted median | 8 | 0.126 |
| genus.DefluviitaleaceaeUCG011.id.11287 | Inverse variance weighted | 8 | 0.263 |
| genus.DefluviitaleaceaeUCG011.id.11287 | Simple mode | 8 | 0.356 |
| genus.DefluviitaleaceaeUCG011.id.11287 | Weighted mode | 8 | 0.296 |
| genus.Coprococcus3.id.11303 | MR Egger | 7 | 0.816 |
| genus.Coprococcus3.id.11303 | Weighted median | 7 | 0.912 |
| genus.Coprococcus3.id.11303 | Inverse variance weighted | 7 | 0.957 |
| genus.Coprococcus3.id.11303 | Simple mode | 7 | 0.973 |
| genus.Coprococcus3.id.11303 | Weighted mode | 7 | 0.971 |
| genus.Coprococcus2.id.11302 | MR Egger | 11 | 0.782 |
| genus.Coprococcus2.id.11302 | Weighted median | 11 | 0.797 |
| genus.Coprococcus2.id.11302 | Inverse variance weighted | 11 | 0.906 |
| genus.Coprococcus2.id.11302 | Simple mode | 11 | 0.944 |
| genus.Coprococcus2.id.11302 | Weighted mode | 11 | 0.925 |
| genus.Coprococcus1.id.11301 | MR Egger | 14 | 0.551 |
| genus.Coprococcus1.id.11301 | Weighted median | 14 | 0.798 |
| genus.Coprococcus1.id.11301 | Inverse variance weighted | 14 | 0.991 |
| genus.Coprococcus1.id.11301 | Simple mode | 14 | 0.492 |
| genus.Coprococcus1.id.11301 | Weighted mode | 14 | 0.959 |
| genus.Coprobacter.id.949 | MR Egger | 14 | 0.558 |
| genus.Coprobacter.id.949 | Weighted median | 14 | 0.390 |
| genus.Coprobacter.id.949 | Inverse variance weighted | 14 | 0.399 |
| genus.Coprobacter.id.949 | Simple mode | 14 | 0.551 |
| genus.Coprobacter.id.949 | Weighted mode | 14 | 0.511 |
| genus.Collinsella.id.815 | MR Egger | 9 | 0.915 |
| genus.Collinsella.id.815 | Weighted median | 9 | 0.319 |
| genus.Collinsella.id.815 | Inverse variance weighted | 9 | 0.769 |
| genus.Collinsella.id.815 | Simple mode | 9 | 0.382 |
| genus.Collinsella.id.815 | Weighted mode | 9 | 0.322 |
| genus.Clostridiumsensustricto1.id.1873 | MR Egger | 9 | 0.552 |
| genus.Clostridiumsensustricto1.id.1873 | Weighted median | 9 | 0.540 |
| genus.Clostridiumsensustricto1.id.1873 | Inverse variance weighted | 9 | 0.623 |
| genus.Clostridiumsensustricto1.id.1873 | Simple mode | 9 | 0.889 |
| genus.Clostridiumsensustricto1.id.1873 | Weighted mode | 9 | 0.461 |
| genus.ChristensenellaceaeR.7group.id.11283 | MR Egger | 9 | 0.058 |
| genus.ChristensenellaceaeR.7group.id.11283 | Weighted median | 9 | 0.332 |
| genus.ChristensenellaceaeR.7group.id.11283 | Inverse variance weighted | 9 | 0.489 |
| genus.ChristensenellaceaeR.7group.id.11283 | Simple mode | 9 | 0.282 |
| genus.ChristensenellaceaeR.7group.id.11283 | Weighted mode | 9 | 0.314 |
| genus.Catenibacterium.id.2153 | MR Egger | 5 | 0.581 |
| genus.Catenibacterium.id.2153 | Weighted median | 5 | 0.060 |
| genus.Catenibacterium.id.2153 | Inverse variance weighted | 5 | 0.064 |
| genus.Catenibacterium.id.2153 | Simple mode | 5 | 0.160 |
| genus.Catenibacterium.id.2153 | Weighted mode | 5 | 0.196 |
| genus.CandidatusSoleaferrea.id.11350 | MR Egger | 13 | 0.157 |
| genus.CandidatusSoleaferrea.id.11350 | Weighted median | 13 | 0.513 |
| genus.CandidatusSoleaferrea.id.11350 | Inverse variance weighted | 13 | 0.349 |
| genus.CandidatusSoleaferrea.id.11350 | Simple mode | 13 | 0.222 |
| genus.CandidatusSoleaferrea.id.11350 | Weighted mode | 13 | 0.193 |
| genus.Butyrivibrio.id.1993 | MR Egger | 14 | 0.880 |
| genus.Butyrivibrio.id.1993 | Weighted median | 14 | 0.839 |
| genus.Butyrivibrio.id.1993 | Inverse variance weighted | 14 | 0.609 |
| genus.Butyrivibrio.id.1993 | Simple mode | 14 | 0.628 |
| genus.Butyrivibrio.id.1993 | Weighted mode | 14 | 0.550 |
| genus.Butyricimonas.id.945 | MR Egger | 18 | 0.586 |
| genus.Butyricimonas.id.945 | Weighted median | 18 | 0.396 |
| genus.Butyricimonas.id.945 | Inverse variance weighted | 18 | 0.255 |
| genus.Butyricimonas.id.945 | Simple mode | 18 | 0.715 |
| genus.Butyricimonas.id.945 | Weighted mode | 18 | 0.715 |
| genus.Butyricicoccus.id.2055 | MR Egger | 8 | 0.364 |
| genus.Butyricicoccus.id.2055 | Weighted median | 8 | 0.783 |
| genus.Butyricicoccus.id.2055 | Inverse variance weighted | 8 | 0.847 |
| genus.Butyricicoccus.id.2055 | Simple mode | 8 | 0.752 |
| genus.Butyricicoccus.id.2055 | Weighted mode | 8 | 0.506 |
| genus.Blautia.id.1992 | MR Egger | 13 | 0.312 |
| genus.Blautia.id.1992 | Weighted median | 13 | 0.357 |
| genus.Blautia.id.1992 | Inverse variance weighted | 13 | 0.033 |
| genus.Blautia.id.1992 | Simple mode | 13 | 0.882 |
| genus.Blautia.id.1992 | Weighted mode | 13 | 0.934 |
| genus.Bilophila.id.3170 | MR Egger | 15 | 0.907 |
| genus.Bilophila.id.3170 | Weighted median | 15 | 0.686 |
| genus.Bilophila.id.3170 | Inverse variance weighted | 15 | 0.961 |
| genus.Bilophila.id.3170 | Simple mode | 15 | 0.752 |
| genus.Bilophila.id.3170 | Weighted mode | 15 | 0.786 |
| genus.Bifidobacterium.id.436 | MR Egger | 17 | 0.886 |
| genus.Bifidobacterium.id.436 | Weighted median | 17 | 0.254 |
| genus.Bifidobacterium.id.436 | Inverse variance weighted | 17 | 0.402 |
| genus.Bifidobacterium.id.436 | Simple mode | 17 | 0.348 |
| genus.Bifidobacterium.id.436 | Weighted mode | 17 | 0.359 |
| genus.Barnesiella.id.944 | MR Egger | 15 | 0.319 |
| genus.Barnesiella.id.944 | Weighted median | 15 | 0.212 |
| genus.Barnesiella.id.944 | Inverse variance weighted | 15 | 0.153 |
| genus.Barnesiella.id.944 | Simple mode | 15 | 0.369 |
| genus.Barnesiella.id.944 | Weighted mode | 15 | 0.360 |
| genus.Bacteroides.id.918 | MR Egger | 10 | 0.870 |
| genus.Bacteroides.id.918 | Weighted median | 10 | 0.761 |
| genus.Bacteroides.id.918 | Inverse variance weighted | 10 | 0.390 |
| genus.Bacteroides.id.918 | Simple mode | 10 | 0.896 |
| genus.Bacteroides.id.918 | Weighted mode | 10 | 0.829 |
| genus.Anaerotruncus.id.2054 | MR Egger | 13 | 0.011 |
| genus.Anaerotruncus.id.2054 | Weighted median | 13 | 0.472 |
| genus.Anaerotruncus.id.2054 | Inverse variance weighted | 13 | 0.545 |
| genus.Anaerotruncus.id.2054 | Simple mode | 13 | 0.473 |
| genus.Anaerotruncus.id.2054 | Weighted mode | 13 | 0.440 |
| genus.Anaerostipes.id.1991 | MR Egger | 13 | 0.853 |
| genus.Anaerostipes.id.1991 | Weighted median | 13 | 0.742 |
| genus.Anaerostipes.id.1991 | Inverse variance weighted | 13 | 0.395 |
| genus.Anaerostipes.id.1991 | Simple mode | 13 | 0.866 |
| genus.Anaerostipes.id.1991 | Weighted mode | 13 | 0.824 |
| genus.Anaerofilum.id.2053 | MR Egger | 9 | 0.159 |
| genus.Anaerofilum.id.2053 | Weighted median | 9 | 0.758 |
| genus.Anaerofilum.id.2053 | Inverse variance weighted | 9 | 0.510 |
| genus.Anaerofilum.id.2053 | Simple mode | 9 | 0.916 |
| genus.Anaerofilum.id.2053 | Weighted mode | 9 | 0.896 |
| genus.Alloprevotella.id.961 | MR Egger | 7 | 0.882 |
| genus.Alloprevotella.id.961 | Weighted median | 7 | 0.462 |
| genus.Alloprevotella.id.961 | Inverse variance weighted | 7 | 0.294 |
| genus.Alloprevotella.id.961 | Simple mode | 7 | 0.827 |
| genus.Alloprevotella.id.961 | Weighted mode | 7 | 0.869 |
| genus.Allisonella.id.2174 | MR Egger | 6 | 0.061 |
| genus.Allisonella.id.2174 | Weighted median | 6 | 0.487 |
| genus.Allisonella.id.2174 | Inverse variance weighted | 6 | 0.855 |
| genus.Allisonella.id.2174 | Simple mode | 6 | 0.374 |
| genus.Allisonella.id.2174 | Weighted mode | 6 | 0.489 |
| genus.Alistipes.id.968 | MR Egger | 13 | 0.941 |
| genus.Alistipes.id.968 | Weighted median | 13 | 0.690 |
| genus.Alistipes.id.968 | Inverse variance weighted | 13 | 0.619 |
| genus.Alistipes.id.968 | Simple mode | 13 | 0.567 |
| genus.Alistipes.id.968 | Weighted mode | 13 | 0.593 |
| genus.Akkermansia.id.4037 | MR Egger | 13 | 0.915 |
| genus.Akkermansia.id.4037 | Weighted median | 13 | 0.760 |
| genus.Akkermansia.id.4037 | Inverse variance weighted | 13 | 0.956 |
| genus.Akkermansia.id.4037 | Simple mode | 13 | 0.782 |
| genus.Akkermansia.id.4037 | Weighted mode | 13 | 0.802 |
| genus.Adlercreutzia.id.812 | MR Egger | 11 | 0.103 |
| genus.Adlercreutzia.id.812 | Weighted median | 11 | 1.000 |
| genus.Adlercreutzia.id.812 | Inverse variance weighted | 11 | 0.720 |
| genus.Adlercreutzia.id.812 | Simple mode | 11 | 0.838 |
| genus.Adlercreutzia.id.812 | Weighted mode | 11 | 0.642 |
| genus.Actinomyces.id.423 | MR Egger | 6 | 0.395 |
| genus.Actinomyces.id.423 | Weighted median | 6 | 0.856 |
| genus.Actinomyces.id.423 | Inverse variance weighted | 6 | 0.815 |
| genus.Actinomyces.id.423 | Simple mode | 6 | 0.498 |
| genus.Actinomyces.id.423 | Weighted mode | 6 | 0.826 |
| genus..Ruminococcustorquesgroup.id.14377 | MR Egger | 13 | 0.537 |
| genus..Ruminococcustorquesgroup.id.14377 | Weighted median | 13 | 0.377 |
| genus..Ruminococcustorquesgroup.id.14377 | Inverse variance weighted | 13 | 0.579 |
| genus..Ruminococcustorquesgroup.id.14377 | Simple mode | 13 | 0.519 |
| genus..Ruminococcustorquesgroup.id.14377 | Weighted mode | 13 | 0.521 |
| genus.Escherichia.Shigella.id.3504 | MR Egger | 15 | 0.317 |
| genus.Escherichia.Shigella.id.3504 | Weighted median | 15 | 0.194 |
| genus.Escherichia.Shigella.id.3504 | Inverse variance weighted | 15 | 0.610 |
| genus.Escherichia.Shigella.id.3504 | Simple mode | 15 | 0.399 |
| genus.Escherichia.Shigella.id.3504 | Weighted mode | 15 | 0.375 |
| genus.ErysipelotrichaceaeUCG003.id.11384 | MR Egger | 18 | 0.517 |
| genus.ErysipelotrichaceaeUCG003.id.11384 | Weighted median | 18 | 0.399 |
| genus.ErysipelotrichaceaeUCG003.id.11384 | Inverse variance weighted | 18 | 0.471 |
| genus.ErysipelotrichaceaeUCG003.id.11384 | Simple mode | 18 | 0.351 |
| genus.ErysipelotrichaceaeUCG003.id.11384 | Weighted mode | 18 | 0.334 |
| genus.Erysipelatoclostridium.id.11381 | MR Egger | 16 | 0.960 |
| genus.Erysipelatoclostridium.id.11381 | Weighted median | 16 | 0.171 |
| genus.Erysipelatoclostridium.id.11381 | Inverse variance weighted | 16 | 0.090 |
| genus.Erysipelatoclostridium.id.11381 | Simple mode | 16 | 0.383 |
| genus.Erysipelatoclostridium.id.11381 | Weighted mode | 16 | 0.373 |
| genus.Enterorhabdus.id.820 | MR Egger | 9 | 0.667 |
| genus.Enterorhabdus.id.820 | Weighted median | 9 | 0.423 |
| genus.Enterorhabdus.id.820 | Inverse variance weighted | 9 | 0.554 |
| genus.Enterorhabdus.id.820 | Simple mode | 9 | 0.268 |
| genus.Enterorhabdus.id.820 | Weighted mode | 9 | 0.369 |
| genus.Oscillibacter.id.2063 | MR Egger | 14 | 0.620 |
| genus.Oscillibacter.id.2063 | Weighted median | 14 | 0.835 |
| genus.Oscillibacter.id.2063 | Inverse variance weighted | 14 | 0.644 |
| genus.Oscillibacter.id.2063 | Simple mode | 14 | 0.977 |
| genus.Oscillibacter.id.2063 | Weighted mode | 14 | 0.991 |
| genus.Olsenella.id.822 | MR Egger | 10 | 0.376 |
| genus.Olsenella.id.822 | Weighted median | 10 | 0.787 |
| genus.Olsenella.id.822 | Inverse variance weighted | 10 | 0.827 |
| genus.Olsenella.id.822 | Simple mode | 10 | 0.443 |
| genus.Olsenella.id.822 | Weighted mode | 10 | 0.528 |
| genus.Odoribacter.id.952 | MR Egger | 8 | 0.091 |
| genus.Odoribacter.id.952 | Weighted median | 8 | 0.235 |
| genus.Odoribacter.id.952 | Inverse variance weighted | 8 | 0.438 |
| genus.Odoribacter.id.952 | Simple mode | 8 | 0.353 |
| genus.Odoribacter.id.952 | Weighted mode | 8 | 0.372 |
| genus.Methanobrevibacter.id.123 | MR Egger | 8 | 0.508 |
| genus.Methanobrevibacter.id.123 | Weighted median | 8 | 0.655 |
| genus.Methanobrevibacter.id.123 | Inverse variance weighted | 8 | 0.643 |
| genus.Methanobrevibacter.id.123 | Simple mode | 8 | 0.620 |
| genus.Methanobrevibacter.id.123 | Weighted mode | 8 | 0.673 |
| genus.Marvinbryantia.id.2005 | MR Egger | 8 | 0.907 |
| genus.Marvinbryantia.id.2005 | Weighted median | 8 | 0.506 |
| genus.Marvinbryantia.id.2005 | Inverse variance weighted | 8 | 0.440 |
| genus.Marvinbryantia.id.2005 | Simple mode | 8 | 0.661 |
| genus.Marvinbryantia.id.2005 | Weighted mode | 8 | 0.681 |
| genus.Lactococcus.id.1851 | MR Egger | 10 | 0.508 |
| genus.Lactococcus.id.1851 | Weighted median | 10 | 0.127 |
| genus.Lactococcus.id.1851 | Inverse variance weighted | 10 | 0.097 |
| genus.Lactococcus.id.1851 | Simple mode | 10 | 0.301 |
| genus.Lactococcus.id.1851 | Weighted mode | 10 | 0.299 |
| genus.Lactobacillus.id.1837 | MR Egger | 10 | 0.410 |
| genus.Lactobacillus.id.1837 | Weighted median | 10 | 0.395 |
| genus.Lactobacillus.id.1837 | Inverse variance weighted | 10 | 0.850 |
| genus.Lactobacillus.id.1837 | Simple mode | 10 | 0.354 |
| genus.Lactobacillus.id.1837 | Weighted mode | 10 | 0.297 |
| genus.LachnospiraceaeUCG010.id.11330 | MR Egger | 10 | 0.447 |
| genus.LachnospiraceaeUCG010.id.11330 | Weighted median | 10 | 0.289 |
| genus.LachnospiraceaeUCG010.id.11330 | Inverse variance weighted | 10 | 0.487 |
| genus.LachnospiraceaeUCG010.id.11330 | Simple mode | 10 | 0.675 |
| genus.LachnospiraceaeUCG010.id.11330 | Weighted mode | 10 | 0.373 |
| genus.LachnospiraceaeUCG008.id.11328 | MR Egger | 12 | 0.104 |
| genus.LachnospiraceaeUCG008.id.11328 | Weighted median | 12 | 0.241 |
| genus.LachnospiraceaeUCG008.id.11328 | Inverse variance weighted | 12 | 0.633 |
| genus.LachnospiraceaeUCG008.id.11328 | Simple mode | 12 | 0.416 |
| genus.LachnospiraceaeUCG008.id.11328 | Weighted mode | 12 | 0.421 |
| genus.LachnospiraceaeUCG004.id.11324 | MR Egger | 15 | 0.409 |
| genus.LachnospiraceaeUCG004.id.11324 | Weighted median | 15 | 0.809 |
| genus.LachnospiraceaeUCG004.id.11324 | Inverse variance weighted | 15 | 0.768 |
| genus.LachnospiraceaeUCG004.id.11324 | Simple mode | 15 | 0.746 |
| genus.LachnospiraceaeUCG004.id.11324 | Weighted mode | 15 | 0.677 |
| genus.LachnospiraceaeUCG001.id.11321 | MR Egger | 15 | 0.776 |
| genus.LachnospiraceaeUCG001.id.11321 | Weighted median | 15 | 0.058 |
| genus.LachnospiraceaeUCG001.id.11321 | Inverse variance weighted | 15 | 0.270 |
| genus.LachnospiraceaeUCG001.id.11321 | Simple mode | 15 | 0.197 |
| genus.LachnospiraceaeUCG001.id.11321 | Weighted mode | 15 | 0.171 |
| genus.LachnospiraceaeNK4A136group.id.11319 | MR Egger | 16 | 0.125 |
| genus.LachnospiraceaeNK4A136group.id.11319 | Weighted median | 16 | 0.811 |
| genus.LachnospiraceaeNK4A136group.id.11319 | Inverse variance weighted | 16 | 0.637 |
| genus.LachnospiraceaeNK4A136group.id.11319 | Simple mode | 16 | 0.733 |
| genus.LachnospiraceaeNK4A136group.id.11319 | Weighted mode | 16 | 0.820 |
| genus.LachnospiraceaeND3007group.id.11317 | MR Egger | 3 | 0.997 |
| genus.LachnospiraceaeND3007group.id.11317 | Weighted median | 3 | 0.699 |
| genus.LachnospiraceaeND3007group.id.11317 | Inverse variance weighted | 3 | 0.715 |
| genus.LachnospiraceaeND3007group.id.11317 | Simple mode | 3 | 0.719 |
| genus.LachnospiraceaeND3007group.id.11317 | Weighted mode | 3 | 0.712 |
| genus.LachnospiraceaeNC2004group.id.11316 | MR Egger | 9 | 0.443 |
| genus.LachnospiraceaeNC2004group.id.11316 | Weighted median | 9 | 0.555 |
| genus.LachnospiraceaeNC2004group.id.11316 | Inverse variance weighted | 9 | 0.307 |
| genus.LachnospiraceaeNC2004group.id.11316 | Simple mode | 9 | 0.579 |
| genus.LachnospiraceaeNC2004group.id.11316 | Weighted mode | 9 | 0.591 |
| genus.LachnospiraceaeFCS020group.id.11314 | MR Egger | 16 | 0.822 |
| genus.LachnospiraceaeFCS020group.id.11314 | Weighted median | 16 | 0.556 |
| genus.LachnospiraceaeFCS020group.id.11314 | Inverse variance weighted | 16 | 0.468 |
| genus.LachnospiraceaeFCS020group.id.11314 | Simple mode | 16 | 0.748 |
| genus.LachnospiraceaeFCS020group.id.11314 | Weighted mode | 16 | 0.716 |
| genus.Lachnospira.id.2004 | MR Egger | 7 | 0.694 |
| genus.Lachnospira.id.2004 | Weighted median | 7 | 0.631 |
| genus.Lachnospira.id.2004 | Inverse variance weighted | 7 | 0.582 |
| genus.Lachnospira.id.2004 | Simple mode | 7 | 0.309 |
| genus.Lachnospira.id.2004 | Weighted mode | 7 | 0.342 |
| genus.Lachnoclostridium.id.11308 | MR Egger | 13 | 0.772 |
| genus.Lachnoclostridium.id.11308 | Weighted median | 13 | 0.425 |
| genus.Lachnoclostridium.id.11308 | Inverse variance weighted | 13 | 0.558 |
| genus.Lachnoclostridium.id.11308 | Simple mode | 13 | 0.378 |
| genus.Lachnoclostridium.id.11308 | Weighted mode | 13 | 0.354 |
| genus.Intestinimonas.id.2062 | MR Egger | 18 | 0.437 |
| genus.Intestinimonas.id.2062 | Weighted median | 18 | 0.442 |
| genus.Intestinimonas.id.2062 | Inverse variance weighted | 18 | 0.195 |
| genus.Intestinimonas.id.2062 | Simple mode | 18 | 0.593 |
| genus.Intestinimonas.id.2062 | Weighted mode | 18 | 0.575 |
| genus.Intestinibacter.id.11345 | MR Egger | 13 | 0.399 |
| genus.Intestinibacter.id.11345 | Weighted median | 13 | 0.308 |
| genus.Intestinibacter.id.11345 | Inverse variance weighted | 13 | 0.371 |
| genus.Intestinibacter.id.11345 | Simple mode | 13 | 0.252 |
| genus.Intestinibacter.id.11345 | Weighted mode | 13 | 0.262 |
| genus.Hungatella.id.11306 | MR Egger | 4 | 0.940 |
| genus.Hungatella.id.11306 | Weighted median | 4 | 0.434 |
| genus.Hungatella.id.11306 | Inverse variance weighted | 4 | 0.965 |
| genus.Hungatella.id.11306 | Simple mode | 4 | 0.478 |
| genus.Hungatella.id.11306 | Weighted mode | 4 | 0.445 |
| genus.Howardella.id.2000 | MR Egger | 9 | 0.991 |
| genus.Howardella.id.2000 | Weighted median | 9 | 0.180 |
| genus.Howardella.id.2000 | Inverse variance weighted | 9 | 0.069 |
| genus.Howardella.id.2000 | Simple mode | 9 | 0.162 |
| genus.Howardella.id.2000 | Weighted mode | 9 | 0.177 |
| genus.Holdemania.id.2157 | MR Egger | 17 | 0.489 |
| genus.Holdemania.id.2157 | Weighted median | 17 | 0.201 |
| genus.Holdemania.id.2157 | Inverse variance weighted | 17 | 0.771 |
| genus.Holdemania.id.2157 | Simple mode | 17 | 0.240 |
| genus.Holdemania.id.2157 | Weighted mode | 17 | 0.256 |
| genus.Holdemanella.id.11393 | MR Egger | 13 | 0.770 |
| genus.Holdemanella.id.11393 | Weighted median | 13 | 0.656 |
| genus.Holdemanella.id.11393 | Inverse variance weighted | 13 | 0.315 |
| genus.Holdemanella.id.11393 | Simple mode | 13 | 0.753 |
| genus.Holdemanella.id.11393 | Weighted mode | 13 | 0.156 |
| genus.Haemophilus.id.3698 | MR Egger | 13 | 0.638 |
| genus.Haemophilus.id.3698 | Weighted median | 13 | 0.643 |
| genus.Haemophilus.id.3698 | Inverse variance weighted | 13 | 0.557 |
| genus.Haemophilus.id.3698 | Simple mode | 13 | 0.869 |
| genus.Haemophilus.id.3698 | Weighted mode | 13 | 0.856 |
| genus.Gordonibacter.id.821 | MR Egger | 14 | 0.558 |
| genus.Gordonibacter.id.821 | Weighted median | 14 | 0.503 |
| genus.Gordonibacter.id.821 | Inverse variance weighted | 14 | 0.935 |
| genus.Gordonibacter.id.821 | Simple mode | 14 | 0.405 |
| genus.Gordonibacter.id.821 | Weighted mode | 14 | 0.434 |
| genus.Fusicatenibacter.id.11305 | MR Egger | 19 | 0.577 |
| genus.Fusicatenibacter.id.11305 | Weighted median | 19 | 0.207 |
| genus.Fusicatenibacter.id.11305 | Inverse variance weighted | 19 | 0.186 |
| genus.Fusicatenibacter.id.11305 | Simple mode | 19 | 0.283 |
| genus.Fusicatenibacter.id.11305 | Weighted mode | 19 | 0.337 |
| genus.Flavonifractor.id.2059 | MR Egger | 8 | 0.474 |
| genus.Flavonifractor.id.2059 | Weighted median | 8 | 0.728 |
| genus.Flavonifractor.id.2059 | Inverse variance weighted | 8 | 0.843 |
| genus.Flavonifractor.id.2059 | Simple mode | 8 | 0.497 |
| genus.Flavonifractor.id.2059 | Weighted mode | 8 | 0.476 |
| genus.FamilyXIIIUCG001.id.11294 | MR Egger | 10 | 0.560 |
| genus.FamilyXIIIUCG001.id.11294 | Weighted median | 10 | 0.302 |
| genus.FamilyXIIIUCG001.id.11294 | Inverse variance weighted | 10 | 0.796 |
| genus.FamilyXIIIUCG001.id.11294 | Simple mode | 10 | 0.417 |
| genus.FamilyXIIIUCG001.id.11294 | Weighted mode | 10 | 0.380 |
| genus.FamilyXIIIAD3011group.id.11293 | MR Egger | 14 | 0.891 |
| genus.FamilyXIIIAD3011group.id.11293 | Weighted median | 14 | 0.967 |
| genus.FamilyXIIIAD3011group.id.11293 | Inverse variance weighted | 14 | 0.677 |
| genus.FamilyXIIIAD3011group.id.11293 | Simple mode | 14 | 0.924 |
| genus.FamilyXIIIAD3011group.id.11293 | Weighted mode | 14 | 0.951 |
| genus.Faecalibacterium.id.2057 | MR Egger | 13 | 0.476 |
| genus.Faecalibacterium.id.2057 | Weighted median | 13 | 0.468 |
| genus.Faecalibacterium.id.2057 | Inverse variance weighted | 13 | 0.269 |
| genus.Faecalibacterium.id.2057 | Simple mode | 13 | 0.697 |
| genus.Faecalibacterium.id.2057 | Weighted mode | 13 | 0.976 |
| genus.Ruminiclostridium6.id.11356 | MR Egger | 15 | 0.183 |
| genus.Ruminiclostridium6.id.11356 | Weighted median | 15 | 0.783 |
| genus.Ruminiclostridium6.id.11356 | Inverse variance weighted | 15 | 0.525 |
| genus.Ruminiclostridium6.id.11356 | Simple mode | 15 | 0.561 |
| genus.Ruminiclostridium6.id.11356 | Weighted mode | 15 | 0.744 |
| genus.Ruminiclostridium5.id.11355 | MR Egger | 14 | 0.556 |
| genus.Ruminiclostridium5.id.11355 | Weighted median | 14 | 0.182 |
| genus.Ruminiclostridium5.id.11355 | Inverse variance weighted | 14 | 0.174 |
| genus.Ruminiclostridium5.id.11355 | Simple mode | 14 | 0.296 |
| genus.Ruminiclostridium5.id.11355 | Weighted mode | 14 | 0.336 |
| genus.Roseburia.id.2012 | MR Egger | 17 | 0.238 |
| genus.Roseburia.id.2012 | Weighted median | 17 | 0.294 |
| genus.Roseburia.id.2012 | Inverse variance weighted | 17 | 0.699 |
| genus.Roseburia.id.2012 | Simple mode | 17 | 0.421 |
| genus.Roseburia.id.2012 | Weighted mode | 17 | 0.257 |
| genus.Romboutsia.id.11347 | MR Egger | 15 | 0.482 |
| genus.Romboutsia.id.11347 | Weighted median | 15 | 0.177 |
| genus.Romboutsia.id.11347 | Inverse variance weighted | 15 | 0.797 |
| genus.Romboutsia.id.11347 | Simple mode | 15 | 0.389 |
| genus.Romboutsia.id.11347 | Weighted mode | 15 | 0.285 |
| genus.RikenellaceaeRC9gutgroup.id.11191 | MR Egger | 11 | 0.915 |
| genus.RikenellaceaeRC9gutgroup.id.11191 | Weighted median | 11 | 0.437 |
| genus.RikenellaceaeRC9gutgroup.id.11191 | Inverse variance weighted | 11 | 0.612 |
| genus.RikenellaceaeRC9gutgroup.id.11191 | Simple mode | 11 | 0.472 |
| genus.RikenellaceaeRC9gutgroup.id.11191 | Weighted mode | 11 | 0.500 |
| genus.Prevotella9.id.11183 | MR Egger | 18 | 0.350 |
| genus.Prevotella9.id.11183 | Weighted median | 18 | 0.125 |
| genus.Prevotella9.id.11183 | Inverse variance weighted | 18 | 0.110 |
| genus.Prevotella9.id.11183 | Simple mode | 18 | 0.132 |
| genus.Prevotella9.id.11183 | Weighted mode | 18 | 0.113 |
| genus.Prevotella7.id.11182 | MR Egger | 11 | 0.998 |
| genus.Prevotella7.id.11182 | Weighted median | 11 | 0.278 |
| genus.Prevotella7.id.11182 | Inverse variance weighted | 11 | 0.131 |
| genus.Prevotella7.id.11182 | Simple mode | 11 | 0.676 |
| genus.Prevotella7.id.11182 | Weighted mode | 11 | 0.623 |
| genus.Phascolarctobacterium.id.2168 | MR Egger | 11 | 0.198 |
| genus.Phascolarctobacterium.id.2168 | Weighted median | 11 | 0.983 |
| genus.Phascolarctobacterium.id.2168 | Inverse variance weighted | 11 | 0.960 |
| genus.Phascolarctobacterium.id.2168 | Simple mode | 11 | 0.389 |
| genus.Phascolarctobacterium.id.2168 | Weighted mode | 11 | 0.382 |
| genus.Peptococcus.id.2037 | MR Egger | 18 | 0.231 |
| genus.Peptococcus.id.2037 | Weighted median | 18 | 0.841 |
| genus.Peptococcus.id.2037 | Inverse variance weighted | 18 | 0.931 |
| genus.Peptococcus.id.2037 | Simple mode | 18 | 0.571 |
| genus.Peptococcus.id.2037 | Weighted mode | 18 | 0.524 |
| genus.Parasutterella.id.2892 | MR Egger | 15 | 0.760 |
| genus.Parasutterella.id.2892 | Weighted median | 15 | 0.662 |
| genus.Parasutterella.id.2892 | Inverse variance weighted | 15 | 0.779 |
| genus.Parasutterella.id.2892 | Simple mode | 15 | 0.792 |
| genus.Parasutterella.id.2892 | Weighted mode | 15 | 0.715 |
| genus.Paraprevotella.id.962 | MR Egger | 11 | 0.939 |
| genus.Paraprevotella.id.962 | Weighted median | 11 | 0.379 |
| genus.Paraprevotella.id.962 | Inverse variance weighted | 11 | 0.445 |
| genus.Paraprevotella.id.962 | Simple mode | 11 | 0.332 |
| genus.Paraprevotella.id.962 | Weighted mode | 11 | 0.455 |
| genus.Parabacteroides.id.954 | MR Egger | 9 | 0.162 |
| genus.Parabacteroides.id.954 | Weighted median | 9 | 0.050 |
| genus.Parabacteroides.id.954 | Inverse variance weighted | 9 | 0.037 |
| genus.Parabacteroides.id.954 | Simple mode | 9 | 0.187 |
| genus.Parabacteroides.id.954 | Weighted mode | 9 | 0.115 |
| genus.Oxalobacter.id.2978 | MR Egger | 11 | 0.273 |
| genus.Oxalobacter.id.2978 | Weighted median | 11 | 0.526 |
| genus.Oxalobacter.id.2978 | Inverse variance weighted | 11 | 0.140 |
| genus.Oxalobacter.id.2978 | Simple mode | 11 | 0.800 |
| genus.Oxalobacter.id.2978 | Weighted mode | 11 | 0.826 |
| genus.Oscillospira.id.2064 | MR Egger | 8 | 0.230 |
| genus.Oscillospira.id.2064 | Weighted median | 8 | 0.951 |
| genus.Oscillospira.id.2064 | Inverse variance weighted | 8 | 0.150 |
| genus.Oscillospira.id.2064 | Simple mode | 8 | 0.932 |
| genus.Oscillospira.id.2064 | Weighted mode | 8 | 0.970 |
| genus.unknowngenus.id.959 | MR Egger | 12 | 0.491 |
| genus.unknowngenus.id.959 | Weighted median | 12 | 0.970 |
| genus.unknowngenus.id.959 | Inverse variance weighted | 12 | 0.695 |
| genus.unknowngenus.id.959 | Simple mode | 12 | 0.428 |
| genus.unknowngenus.id.959 | Weighted mode | 12 | 0.598 |
| genus.unknowngenus.id.826 | MR Egger | 15 | 0.911 |
| genus.unknowngenus.id.826 | Weighted median | 15 | 0.759 |
| genus.unknowngenus.id.826 | Inverse variance weighted | 15 | 0.938 |
| genus.unknowngenus.id.826 | Simple mode | 15 | 0.935 |
| genus.unknowngenus.id.826 | Weighted mode | 15 | 0.870 |
| genus.unknowngenus.id.2755 | MR Egger | 15 | 0.114 |
| genus.unknowngenus.id.2755 | Weighted median | 15 | 0.682 |
| genus.unknowngenus.id.2755 | Inverse variance weighted | 15 | 0.795 |
| genus.unknowngenus.id.2755 | Simple mode | 15 | 0.325 |
| genus.unknowngenus.id.2755 | Weighted mode | 15 | 0.418 |
| genus.unknowngenus.id.2071 | MR Egger | 20 | 0.098 |
| genus.unknowngenus.id.2071 | Weighted median | 20 | 0.848 |
| genus.unknowngenus.id.2071 | Inverse variance weighted | 20 | 0.618 |
| genus.unknowngenus.id.2071 | Simple mode | 20 | 0.474 |
| genus.unknowngenus.id.2071 | Weighted mode | 20 | 0.499 |
| genus.unknowngenus.id.2041 | MR Egger | 12 | 0.615 |
| genus.unknowngenus.id.2041 | Weighted median | 12 | 0.867 |
| genus.unknowngenus.id.2041 | Inverse variance weighted | 12 | 0.448 |
| genus.unknowngenus.id.2041 | Simple mode | 12 | 0.898 |
| genus.unknowngenus.id.2041 | Weighted mode | 12 | 0.857 |
| genus.unknowngenus.id.2001 | MR Egger | 11 | 0.315 |
| genus.unknowngenus.id.2001 | Weighted median | 11 | 0.481 |
| genus.unknowngenus.id.2001 | Inverse variance weighted | 11 | 0.264 |
| genus.unknowngenus.id.2001 | Simple mode | 11 | 0.998 |
| genus.unknowngenus.id.2001 | Weighted mode | 11 | 0.991 |
| genus.unknowngenus.id.1868 | MR Egger | 11 | 0.253 |
| genus.unknowngenus.id.1868 | Weighted median | 11 | 0.839 |
| genus.unknowngenus.id.1868 | Inverse variance weighted | 11 | 0.945 |
| genus.unknowngenus.id.1868 | Simple mode | 11 | 0.701 |
| genus.unknowngenus.id.1868 | Weighted mode | 11 | 0.701 |
| genus.unknowngenus.id.1000006162 | MR Egger | 15 | 0.458 |
| genus.unknowngenus.id.1000006162 | Weighted median | 15 | 0.083 |
| genus.unknowngenus.id.1000006162 | Inverse variance weighted | 15 | 0.072 |
| genus.unknowngenus.id.1000006162 | Simple mode | 15 | 0.200 |
| genus.unknowngenus.id.1000006162 | Weighted mode | 15 | 0.229 |
| genus.unknowngenus.id.1000005479 | MR Egger | 9 | 0.604 |
| genus.unknowngenus.id.1000005479 | Weighted median | 9 | 0.840 |
| genus.unknowngenus.id.1000005479 | Inverse variance weighted | 9 | 0.883 |
| genus.unknowngenus.id.1000005479 | Simple mode | 9 | 0.833 |
| genus.unknowngenus.id.1000005479 | Weighted mode | 9 | 0.826 |
| genus.unknowngenus.id.1000005472 | MR Egger | 14 | 0.614 |
| genus.unknowngenus.id.1000005472 | Weighted median | 14 | 0.880 |
| genus.unknowngenus.id.1000005472 | Inverse variance weighted | 14 | 0.245 |
| genus.unknowngenus.id.1000005472 | Simple mode | 14 | 0.563 |
| genus.unknowngenus.id.1000005472 | Weighted mode | 14 | 0.452 |
| genus.unknowngenus.id.1000001215 | MR Egger | 11 | 0.726 |
| genus.unknowngenus.id.1000001215 | Weighted median | 11 | 0.579 |
| genus.unknowngenus.id.1000001215 | Inverse variance weighted | 11 | 0.139 |
| genus.unknowngenus.id.1000001215 | Simple mode | 11 | 0.759 |
| genus.unknowngenus.id.1000001215 | Weighted mode | 11 | 0.692 |
| genus.unknowngenus.id.1000000073 | MR Egger | 15 | 0.934 |
| genus.unknowngenus.id.1000000073 | Weighted median | 15 | 0.894 |
| genus.unknowngenus.id.1000000073 | Inverse variance weighted | 15 | 0.839 |
| genus.unknowngenus.id.1000000073 | Simple mode | 15 | 0.815 |
| genus.unknowngenus.id.1000000073 | Weighted mode | 15 | 0.848 |
| genus.Tyzzerella3.id.11335 | MR Egger | 13 | 0.909 |
| genus.Tyzzerella3.id.11335 | Weighted median | 13 | 0.413 |
| genus.Tyzzerella3.id.11335 | Inverse variance weighted | 13 | 0.120 |
| genus.Tyzzerella3.id.11335 | Simple mode | 13 | 0.418 |
| genus.Tyzzerella3.id.11335 | Weighted mode | 13 | 0.507 |
| genus.Turicibacter.id.2162 | MR Egger | 13 | 0.303 |
| genus.Turicibacter.id.2162 | Weighted median | 13 | 0.653 |
| genus.Turicibacter.id.2162 | Inverse variance weighted | 13 | 0.910 |
| genus.Turicibacter.id.2162 | Simple mode | 13 | 0.603 |
| genus.Turicibacter.id.2162 | Weighted mode | 13 | 0.639 |
| genus.Terrisporobacter.id.11348 | MR Egger | 5 | 0.766 |
| genus.Terrisporobacter.id.11348 | Weighted median | 5 | 0.172 |
| genus.Terrisporobacter.id.11348 | Inverse variance weighted | 5 | 0.371 |
| genus.Terrisporobacter.id.11348 | Simple mode | 5 | 0.194 |
| genus.Terrisporobacter.id.11348 | Weighted mode | 5 | 0.324 |
| genus.Sutterella.id.2896 | MR Egger | 12 | 0.949 |
| genus.Sutterella.id.2896 | Weighted median | 12 | 0.292 |
| genus.Sutterella.id.2896 | Inverse variance weighted | 12 | 0.307 |
| genus.Sutterella.id.2896 | Simple mode | 12 | 0.462 |
| genus.Sutterella.id.2896 | Weighted mode | 12 | 0.475 |
| genus.Subdoligranulum.id.2070 | MR Egger | 13 | 0.242 |
| genus.Subdoligranulum.id.2070 | Weighted median | 13 | 0.890 |
| genus.Subdoligranulum.id.2070 | Inverse variance weighted | 13 | 0.581 |
| genus.Subdoligranulum.id.2070 | Simple mode | 13 | 0.875 |
| genus.Subdoligranulum.id.2070 | Weighted mode | 13 | 0.961 |
| genus.Streptococcus.id.1853 | MR Egger | 17 | 0.665 |
| genus.Streptococcus.id.1853 | Weighted median | 17 | 0.367 |
| genus.Streptococcus.id.1853 | Inverse variance weighted | 17 | 0.642 |
| genus.Streptococcus.id.1853 | Simple mode | 17 | 0.428 |
| genus.Streptococcus.id.1853 | Weighted mode | 17 | 0.386 |
| genus.Slackia.id.825 | MR Egger | 9 | 0.039 |
| genus.Slackia.id.825 | Weighted median | 9 | 0.340 |
| genus.Slackia.id.825 | Inverse variance weighted | 9 | 0.121 |
| genus.Slackia.id.825 | Simple mode | 9 | 0.510 |
| genus.Slackia.id.825 | Weighted mode | 9 | 0.521 |
| genus.Senegalimassilia.id.11160 | MR Egger | 5 | 0.535 |
| genus.Senegalimassilia.id.11160 | Weighted median | 5 | 0.120 |
| genus.Senegalimassilia.id.11160 | Inverse variance weighted | 5 | 0.198 |
| genus.Senegalimassilia.id.11160 | Simple mode | 5 | 0.307 |
| genus.Senegalimassilia.id.11160 | Weighted mode | 5 | 0.308 |
| genus.Sellimonas.id.14369 | MR Egger | 12 | 0.757 |
| genus.Sellimonas.id.14369 | Weighted median | 12 | 0.306 |
| genus.Sellimonas.id.14369 | Inverse variance weighted | 12 | 0.679 |
| genus.Sellimonas.id.14369 | Simple mode | 12 | 0.382 |
| genus.Sellimonas.id.14369 | Weighted mode | 12 | 0.455 |
| genus.Ruminococcus2.id.11374 | MR Egger | 14 | 0.892 |
| genus.Ruminococcus2.id.11374 | Weighted median | 14 | 0.353 |
| genus.Ruminococcus2.id.11374 | Inverse variance weighted | 14 | 0.470 |
| genus.Ruminococcus2.id.11374 | Simple mode | 14 | 0.501 |
| genus.Ruminococcus2.id.11374 | Weighted mode | 14 | 0.506 |
| genus.Ruminococcus1.id.11373 | MR Egger | 13 | 0.845 |
| genus.Ruminococcus1.id.11373 | Weighted median | 13 | 0.888 |
| genus.Ruminococcus1.id.11373 | Inverse variance weighted | 13 | 0.917 |
| genus.Ruminococcus1.id.11373 | Simple mode | 13 | 0.961 |
| genus.Ruminococcus1.id.11373 | Weighted mode | 13 | 0.850 |
| genus.RuminococcaceaeUCG014.id.11371 | MR Egger | 18 | 0.372 |
| genus.RuminococcaceaeUCG014.id.11371 | Weighted median | 18 | 0.739 |
| genus.RuminococcaceaeUCG014.id.11371 | Inverse variance weighted | 18 | 0.959 |
| genus.RuminococcaceaeUCG014.id.11371 | Simple mode | 18 | 0.175 |
| genus.RuminococcaceaeUCG014.id.11371 | Weighted mode | 18 | 0.585 |
| genus.RuminococcaceaeUCG013.id.11370 | MR Egger | 12 | 0.152 |
| genus.RuminococcaceaeUCG013.id.11370 | Weighted median | 12 | 0.011 |
| genus.RuminococcaceaeUCG013.id.11370 | Inverse variance weighted | 12 | 0.104 |
| genus.RuminococcaceaeUCG013.id.11370 | Simple mode | 12 | 0.170 |
| genus.RuminococcaceaeUCG013.id.11370 | Weighted mode | 12 | 0.092 |
| genus.RuminococcaceaeUCG011.id.11368 | MR Egger | 8 | 0.436 |
| genus.RuminococcaceaeUCG011.id.11368 | Weighted median | 8 | 0.261 |
| genus.RuminococcaceaeUCG011.id.11368 | Inverse variance weighted | 8 | 0.137 |
| genus.RuminococcaceaeUCG011.id.11368 | Simple mode | 8 | 0.407 |
| genus.RuminococcaceaeUCG011.id.11368 | Weighted mode | 8 | 0.431 |
| genus.RuminococcaceaeUCG010.id.11367 | MR Egger | 7 | 0.660 |
| genus.RuminococcaceaeUCG010.id.11367 | Weighted median | 7 | 0.396 |
| genus.RuminococcaceaeUCG010.id.11367 | Inverse variance weighted | 7 | 0.242 |
| genus.RuminococcaceaeUCG010.id.11367 | Simple mode | 7 | 0.582 |
| genus.RuminococcaceaeUCG010.id.11367 | Weighted mode | 7 | 0.737 |
| genus.RuminococcaceaeUCG009.id.11366 | MR Egger | 13 | 0.810 |
| genus.RuminococcaceaeUCG009.id.11366 | Weighted median | 13 | 0.355 |
| genus.RuminococcaceaeUCG009.id.11366 | Inverse variance weighted | 13 | 0.545 |
| genus.RuminococcaceaeUCG009.id.11366 | Simple mode | 13 | 0.232 |
| genus.RuminococcaceaeUCG009.id.11366 | Weighted mode | 13 | 0.254 |
| genus.RuminococcaceaeUCG005.id.11363 | MR Egger | 17 | 0.565 |
| genus.RuminococcaceaeUCG005.id.11363 | Weighted median | 17 | 0.732 |
| genus.RuminococcaceaeUCG005.id.11363 | Inverse variance weighted | 17 | 0.718 |
| genus.RuminococcaceaeUCG005.id.11363 | Simple mode | 17 | 0.644 |
| genus.RuminococcaceaeUCG005.id.11363 | Weighted mode | 17 | 0.927 |
| genus.RuminococcaceaeUCG004.id.11362 | MR Egger | 9 | 0.316 |
| genus.RuminococcaceaeUCG004.id.11362 | Weighted median | 9 | 0.273 |
| genus.RuminococcaceaeUCG004.id.11362 | Inverse variance weighted | 9 | 0.906 |
| genus.RuminococcaceaeUCG004.id.11362 | Simple mode | 9 | 0.353 |
| genus.RuminococcaceaeUCG004.id.11362 | Weighted mode | 9 | 0.342 |
| genus.RuminococcaceaeUCG003.id.11361 | MR Egger | 13 | 0.535 |
| genus.RuminococcaceaeUCG003.id.11361 | Weighted median | 13 | 0.251 |
| genus.RuminococcaceaeUCG003.id.11361 | Inverse variance weighted | 13 | 0.208 |
| genus.RuminococcaceaeUCG003.id.11361 | Simple mode | 13 | 0.447 |
| genus.RuminococcaceaeUCG003.id.11361 | Weighted mode | 13 | 0.427 |
| genus.RuminococcaceaeUCG002.id.11360 | MR Egger | 24 | 0.507 |
| genus.RuminococcaceaeUCG002.id.11360 | Weighted median | 24 | 0.529 |
| genus.RuminococcaceaeUCG002.id.11360 | Inverse variance weighted | 24 | 0.365 |
| genus.RuminococcaceaeUCG002.id.11360 | Simple mode | 24 | 0.104 |
| genus.RuminococcaceaeUCG002.id.11360 | Weighted mode | 24 | 0.533 |
| genus.RuminococcaceaeNK4A214group.id.11358 | MR Egger | 14 | 0.157 |
| genus.RuminococcaceaeNK4A214group.id.11358 | Weighted median | 14 | 0.606 |
| genus.RuminococcaceaeNK4A214group.id.11358 | Inverse variance weighted | 14 | 0.485 |
| genus.RuminococcaceaeNK4A214group.id.11358 | Simple mode | 14 | 0.522 |
| genus.RuminococcaceaeNK4A214group.id.11358 | Weighted mode | 14 | 0.501 |
| genus.Ruminiclostridium9.id.11357 | MR Egger | 14 | 0.855 |
| genus.Ruminiclostridium9.id.11357 | Weighted median | 14 | 0.978 |
| genus.Ruminiclostridium9.id.11357 | Inverse variance weighted | 14 | 0.956 |
| genus.Ruminiclostridium9.id.11357 | Simple mode | 14 | 0.661 |
| genus.Ruminiclostridium9.id.11357 | Weighted mode | 14 | 0.799 |
| phylum.Verrucomicrobia.id.3982 | MR Egger | 12 | 0.819 |
| phylum.Verrucomicrobia.id.3982 | Weighted median | 12 | 0.572 |
| phylum.Verrucomicrobia.id.3982 | Inverse variance weighted | 12 | 0.766 |
| phylum.Verrucomicrobia.id.3982 | Simple mode | 12 | 0.702 |
| phylum.Verrucomicrobia.id.3982 | Weighted mode | 12 | 0.786 |
| phylum.Tenericutes.id.3919 | MR Egger | 12 | 0.340 |
| phylum.Tenericutes.id.3919 | Weighted median | 12 | 0.911 |
| phylum.Tenericutes.id.3919 | Inverse variance weighted | 12 | 0.537 |
| phylum.Tenericutes.id.3919 | Simple mode | 12 | 0.400 |
| phylum.Tenericutes.id.3919 | Weighted mode | 12 | 0.579 |
| phylum.Proteobacteria.id.2375 | MR Egger | 14 | 0.122 |
| phylum.Proteobacteria.id.2375 | Weighted median | 14 | 0.655 |
| phylum.Proteobacteria.id.2375 | Inverse variance weighted | 14 | 0.299 |
| phylum.Proteobacteria.id.2375 | Simple mode | 14 | 0.849 |
| phylum.Proteobacteria.id.2375 | Weighted mode | 14 | 0.861 |
| phylum.Lentisphaerae.id.2238 | MR Egger | 11 | 0.783 |
| phylum.Lentisphaerae.id.2238 | Weighted median | 11 | 0.195 |
| phylum.Lentisphaerae.id.2238 | Inverse variance weighted | 11 | 0.467 |
| phylum.Lentisphaerae.id.2238 | Simple mode | 11 | 0.139 |
| phylum.Lentisphaerae.id.2238 | Weighted mode | 11 | 0.187 |
| phylum.Firmicutes.id.1672 | MR Egger | 19 | 0.070 |
| phylum.Firmicutes.id.1672 | Weighted median | 19 | 0.319 |
| phylum.Firmicutes.id.1672 | Inverse variance weighted | 19 | 0.452 |
| phylum.Firmicutes.id.1672 | Simple mode | 19 | 0.356 |
| phylum.Firmicutes.id.1672 | Weighted mode | 19 | 0.512 |
| phylum.Euryarchaeota.id.55 | MR Egger | 13 | 0.048 |
| phylum.Euryarchaeota.id.55 | Weighted median | 13 | 0.977 |
| phylum.Euryarchaeota.id.55 | Inverse variance weighted | 13 | 0.527 |
| phylum.Euryarchaeota.id.55 | Simple mode | 13 | 0.983 |
| phylum.Euryarchaeota.id.55 | Weighted mode | 13 | 0.982 |
| phylum.Cyanobacteria.id.1500 | MR Egger | 9 | 0.299 |
| phylum.Cyanobacteria.id.1500 | Weighted median | 9 | 0.040 |
| phylum.Cyanobacteria.id.1500 | Inverse variance weighted | 9 | 0.044 |
| phylum.Cyanobacteria.id.1500 | Simple mode | 9 | 0.181 |
| phylum.Cyanobacteria.id.1500 | Weighted mode | 9 | 0.164 |
| phylum.Bacteroidetes.id.905 | MR Egger | 14 | 0.569 |
| phylum.Bacteroidetes.id.905 | Weighted median | 14 | 0.097 |
| phylum.Bacteroidetes.id.905 | Inverse variance weighted | 14 | 0.054 |
| phylum.Bacteroidetes.id.905 | Simple mode | 14 | 0.418 |
| phylum.Bacteroidetes.id.905 | Weighted mode | 14 | 0.247 |
| phylum.Actinobacteria.id.400 | MR Egger | 17 | 0.845 |
| phylum.Actinobacteria.id.400 | Weighted median | 17 | 0.249 |
| phylum.Actinobacteria.id.400 | Inverse variance weighted | 17 | 0.487 |
| phylum.Actinobacteria.id.400 | Simple mode | 17 | 0.600 |
| phylum.Actinobacteria.id.400 | Weighted mode | 17 | 0.416 |
| order.Victivallales.id.2254 | MR Egger | 10 | 0.767 |
| order.Victivallales.id.2254 | Weighted median | 10 | 0.158 |
| order.Victivallales.id.2254 | Inverse variance weighted | 10 | 0.491 |
| order.Victivallales.id.2254 | Simple mode | 10 | 0.143 |
| order.Victivallales.id.2254 | Weighted mode | 10 | 0.198 |
| order.Verrucomicrobiales.id.4030 | MR Egger | 13 | 0.916 |
| order.Verrucomicrobiales.id.4030 | Weighted median | 13 | 0.765 |
| order.Verrucomicrobiales.id.4030 | Inverse variance weighted | 13 | 0.956 |
| order.Verrucomicrobiales.id.4030 | Simple mode | 13 | 0.787 |
| order.Verrucomicrobiales.id.4030 | Weighted mode | 13 | 0.811 |
| order.Selenomonadales.id.2165 | MR Egger | 12 | 0.372 |
| order.Selenomonadales.id.2165 | Weighted median | 12 | 0.977 |
| order.Selenomonadales.id.2165 | Inverse variance weighted | 12 | 0.784 |
| order.Selenomonadales.id.2165 | Simple mode | 12 | 0.527 |
| order.Selenomonadales.id.2165 | Weighted mode | 12 | 0.667 |
| order.Rhodospirillales.id.2667 | MR Egger | 15 | 0.135 |
| order.Rhodospirillales.id.2667 | Weighted median | 15 | 0.651 |
| order.Rhodospirillales.id.2667 | Inverse variance weighted | 15 | 0.724 |
| order.Rhodospirillales.id.2667 | Simple mode | 15 | 0.548 |
| order.Rhodospirillales.id.2667 | Weighted mode | 15 | 0.559 |
| order.Pasteurellales.id.3688 | MR Egger | 17 | 0.451 |
| order.Pasteurellales.id.3688 | Weighted median | 17 | 0.818 |
| order.Pasteurellales.id.3688 | Inverse variance weighted | 17 | 0.605 |
| order.Pasteurellales.id.3688 | Simple mode | 17 | 0.882 |
| order.Pasteurellales.id.3688 | Weighted mode | 17 | 0.808 |
| order.NB1n.id.3953 | MR Egger | 15 | 0.458 |
| order.NB1n.id.3953 | Weighted median | 15 | 0.093 |
| order.NB1n.id.3953 | Inverse variance weighted | 15 | 0.072 |
| order.NB1n.id.3953 | Simple mode | 15 | 0.232 |
| order.NB1n.id.3953 | Weighted mode | 15 | 0.232 |
| order.MollicutesRF9.id.11579 | MR Egger | 14 | 0.614 |
| order.MollicutesRF9.id.11579 | Weighted median | 14 | 0.878 |
| order.MollicutesRF9.id.11579 | Inverse variance weighted | 14 | 0.245 |
| order.MollicutesRF9.id.11579 | Simple mode | 14 | 0.554 |
| order.MollicutesRF9.id.11579 | Weighted mode | 14 | 0.436 |
| order.Methanobacteriales.id.120 | MR Egger | 12 | 0.160 |
| order.Methanobacteriales.id.120 | Weighted median | 12 | 0.265 |
| order.Methanobacteriales.id.120 | Inverse variance weighted | 12 | 0.552 |
| order.Methanobacteriales.id.120 | Simple mode | 12 | 0.543 |
| order.Methanobacteriales.id.120 | Weighted mode | 12 | 0.479 |
| order.Lactobacillales.id.1800 | MR Egger | 17 | 0.905 |
| order.Lactobacillales.id.1800 | Weighted median | 17 | 0.975 |
| order.Lactobacillales.id.1800 | Inverse variance weighted | 17 | 0.920 |
| order.Lactobacillales.id.1800 | Simple mode | 17 | 0.892 |
| order.Lactobacillales.id.1800 | Weighted mode | 17 | 0.976 |
| order.Gastranaerophilales.id.1591 | MR Egger | 11 | 0.726 |
| order.Gastranaerophilales.id.1591 | Weighted median | 11 | 0.588 |
| order.Gastranaerophilales.id.1591 | Inverse variance weighted | 11 | 0.139 |
| order.Gastranaerophilales.id.1591 | Simple mode | 11 | 0.743 |
| order.Gastranaerophilales.id.1591 | Weighted mode | 11 | 0.678 |
| order.Erysipelotrichales.id.2148 | MR Egger | 13 | 0.084 |
| order.Erysipelotrichales.id.2148 | Weighted median | 13 | 0.618 |
| order.Erysipelotrichales.id.2148 | Inverse variance weighted | 13 | 0.664 |
| order.Erysipelotrichales.id.2148 | Simple mode | 13 | 0.213 |
| order.Erysipelotrichales.id.2148 | Weighted mode | 13 | 0.217 |
| order.Enterobacteriales.id.3468 | MR Egger | 10 | 0.475 |
| order.Enterobacteriales.id.3468 | Weighted median | 10 | 0.428 |
| order.Enterobacteriales.id.3468 | Inverse variance weighted | 10 | 0.230 |
| order.Enterobacteriales.id.3468 | Simple mode | 10 | 0.639 |
| order.Enterobacteriales.id.3468 | Weighted mode | 10 | 0.616 |
| order.Desulfovibrionales.id.3156 | MR Egger | 13 | 0.218 |
| order.Desulfovibrionales.id.3156 | Weighted median | 13 | 0.562 |
| order.Desulfovibrionales.id.3156 | Inverse variance weighted | 13 | 0.663 |
| order.Desulfovibrionales.id.3156 | Simple mode | 13 | 0.436 |
| order.Desulfovibrionales.id.3156 | Weighted mode | 13 | 0.270 |
| order.Coriobacteriales.id.810 | MR Egger | 19 | 0.747 |
| order.Coriobacteriales.id.810 | Weighted median | 19 | 0.824 |
| order.Coriobacteriales.id.810 | Inverse variance weighted | 19 | 0.980 |
| order.Coriobacteriales.id.810 | Simple mode | 19 | 0.766 |
| order.Coriobacteriales.id.810 | Weighted mode | 19 | 0.975 |
| order.Clostridiales.id.1863 | MR Egger | 16 | 0.348 |
| order.Clostridiales.id.1863 | Weighted median | 16 | 0.346 |
| order.Clostridiales.id.1863 | Inverse variance weighted | 16 | 0.720 |
| order.Clostridiales.id.1863 | Simple mode | 16 | 0.442 |
| order.Clostridiales.id.1863 | Weighted mode | 16 | 0.459 |
| order.Burkholderiales.id.2874 | MR Egger | 13 | 0.894 |
| order.Burkholderiales.id.2874 | Weighted median | 13 | 0.648 |
| order.Burkholderiales.id.2874 | Inverse variance weighted | 13 | 0.728 |
| order.Burkholderiales.id.2874 | Simple mode | 13 | 0.311 |
| order.Burkholderiales.id.2874 | Weighted mode | 13 | 0.374 |
| order.Bifidobacteriales.id.432 | MR Egger | 21 | 0.553 |
| order.Bifidobacteriales.id.432 | Weighted median | 21 | 0.286 |
| order.Bifidobacteriales.id.432 | Inverse variance weighted | 21 | 0.325 |
| order.Bifidobacteriales.id.432 | Simple mode | 21 | 0.597 |
| order.Bifidobacteriales.id.432 | Weighted mode | 21 | 0.420 |
| order.Bacteroidales.id.913 | MR Egger | 15 | 0.833 |
| order.Bacteroidales.id.913 | Weighted median | 15 | 0.101 |
| order.Bacteroidales.id.913 | Inverse variance weighted | 15 | 0.027 |
| order.Bacteroidales.id.913 | Simple mode | 15 | 0.195 |
| order.Bacteroidales.id.913 | Weighted mode | 15 | 0.204 |
| order.Bacillales.id.1674 | MR Egger | 11 | 0.017 |
| order.Bacillales.id.1674 | Weighted median | 11 | 0.670 |
| order.Bacillales.id.1674 | Inverse variance weighted | 11 | 0.166 |
| order.Bacillales.id.1674 | Simple mode | 11 | 0.922 |
| order.Bacillales.id.1674 | Weighted mode | 11 | 0.866 |
| order.Actinomycetales.id.420 | MR Egger | 5 | 0.616 |
| order.Actinomycetales.id.420 | Weighted median | 5 | 0.463 |
| order.Actinomycetales.id.420 | Inverse variance weighted | 5 | 0.235 |
| order.Actinomycetales.id.420 | Simple mode | 5 | 0.385 |
| order.Actinomycetales.id.420 | Weighted mode | 5 | 0.532 |
| genus.Victivallis.id.2256 | MR Egger | 12 | 0.823 |
| genus.Victivallis.id.2256 | Weighted median | 12 | 0.133 |
| genus.Victivallis.id.2256 | Inverse variance weighted | 12 | 0.368 |
| genus.Victivallis.id.2256 | Simple mode | 12 | 0.193 |
| genus.Victivallis.id.2256 | Weighted mode | 12 | 0.168 |
| genus.Veillonella.id.2198 | MR Egger | 9 | 0.408 |
| genus.Veillonella.id.2198 | Weighted median | 9 | 0.100 |
| genus.Veillonella.id.2198 | Inverse variance weighted | 9 | 0.046 |
| genus.Veillonella.id.2198 | Simple mode | 9 | 0.209 |
| genus.Veillonella.id.2198 | Weighted mode | 9 | 0.257 |

Table S2 Characteristics of six gut microbiota associated SNPs associated with the risk of CM.

| Group | Gut microbiota | SNP | Beta | SE | *F* |
| --- | --- | --- | --- | --- | --- |
| class | *Bacteroidia* | rs11146701 | 0.047 | 0.011 | 20.184 |
|  |  | rs111845179 | 0.103 | 0.021 | 23.039 |
|  |  | rs13291169 | 0.069 | 0.015 | 21.490 |
|  |  | rs17343978 | -0.055 | 0.012 | 21.064 |
|  |  | rs2032750 | -0.051 | 0.011 | 22.654 |
|  |  | rs4146051 | -0.107 | 0.025 | 19.102 |
|  |  | rs55773148 | -0.122 | 0.024 | 26.337 |
|  |  | rs62531359 | 0.066 | 0.015 | 19.136 |
|  |  | rs62575403 | 0.140 | 0.031 | 20.259 |
|  |  | rs72706335 | -0.222 | 0.049 | 20.304 |
|  |  | rs73975615 | -0.207 | 0.044 | 21.861 |
|  |  | rs7546249 | -0.057 | 0.012 | 22.945 |
|  |  | rs7631304 | -0.065 | 0.013 | 23.588 |
|  |  | rs79585701 | 0.065 | 0.015 | 18.685 |
|  |  | rs929878 | -0.055 | 0.012 | 20.370 |
| order | *Bacteroidales* | rs11146701 | 0.047 | 0.011 | 20.184 |
|  |  | rs111845179 | 0.103 | 0.021 | 23.039 |
|  |  | rs13291169 | 0.069 | 0.015 | 21.490 |
|  |  | rs17343978 | -0.055 | 0.012 | 21.064 |
|  |  | rs2032750 | -0.051 | 0.011 | 22.654 |
|  |  | rs4146051 | -0.107 | 0.025 | 19.102 |
|  |  | rs55773148 | -0.122 | 0.024 | 26.337 |
|  |  | rs62531359 | 0.066 | 0.015 | 19.136 |
|  |  | rs62575403 | 0.140 | 0.031 | 20.259 |
|  |  | rs72706335 | -0.222 | 0.049 | 20.304 |
|  |  | rs73975615 | -0.207 | 0.044 | 21.861 |
|  |  | rs7546249 | -0.057 | 0.012 | 22.945 |
|  |  | rs7631304 | -0.065 | 0.013 | 23.588 |
|  |  | rs79585701 | 0.065 | 0.015 | 18.685 |
|  |  | rs929878 | -0.055 | 0.012 | 20.370 |
| genus | *Parabacteroides* | rs114567323 | 0.186 | 0.041 | 21.172 |
|  |  | rs115602804 | 0.103 | 0.022 | 21.415 |
|  |  | rs17141986 | 0.050 | 0.011 | 20.146 |
|  |  | rs3860755 | 0.056 | 0.012 | 22.954 |
|  |  | rs4236095 | 0.076 | 0.016 | 23.539 |
|  |  | rs60884758 | -0.070 | 0.014 | 24.398 |
|  |  | rs6657302 | -0.105 | 0.023 | 21.477 |
|  |  | rs72893646 | -0.072 | 0.016 | 20.860 |
|  |  | rs7298818 | 0.089 | 0.020 | 19.571 |
| genus | *Veillonella* | rs12679709 | -0.079 | 0.016 | 23.173 |
|  |  | rs1882878 | -0.077 | 0.016 | 22.005 |
|  |  | rs2013594 | -0.072 | 0.016 | 21.572 |
|  |  | rs55807413 | 0.107 | 0.024 | 20.395 |
|  |  | rs62376424 | -0.076 | 0.016 | 21.728 |
|  |  | rs6656807 | 0.070 | 0.015 | 20.850 |
|  |  | rs7359080 | -0.135 | 0.030 | 19.891 |
|  |  | rs742016 | -0.069 | 0.015 | 21.133 |
|  |  | rs7645873 | 0.076 | 0.016 | 21.489 |
| genus | *Blautia* | rs11149971 | 0.118 | 0.023 | 25.278 |
|  |  | rs113271346 | 0.078 | 0.017 | 20.676 |
|  |  | rs115043014 | -0.207 | 0.044 | 22.048 |
|  |  | rs117001700 | 0.196 | 0.044 | 19.818 |
|  |  | rs12453000 | 0.063 | 0.013 | 23.141 |
|  |  | rs16892041 | -0.062 | 0.014 | 19.275 |
|  |  | rs2788271 | -0.058 | 0.013 | 18.590 |
|  |  | rs3005511 | 0.050 | 0.011 | 20.462 |
|  |  | rs4926264 | 0.083 | 0.018 | 21.484 |
|  |  | rs67794373 | 0.060 | 0.012 | 23.757 |
|  |  | rs682885 | -0.049 | 0.011 | 21.117 |
|  |  | rs72973581 | 0.125 | 0.027 | 22.238 |
|  |  | rs7860714 | -0.050 | 0.011 | 20.895 |
| phylum | *Cyanobacteria* | rs12555298 | 0.097 | 0.022 | 19.725 |
|  |  | rs2314810 | -0.218 | 0.046 | 22.060 |
|  |  | rs2553290 | 0.096 | 0.020 | 22.542 |
|  |  | rs2585223 | 0.111 | 0.025 | 20.201 |
|  |  | rs584122 | -0.152 | 0.033 | 21.559 |
|  |  | rs61972390 | 0.107 | 0.024 | 19.594 |
|  |  | rs7148504 | 0.080 | 0.018 | 20.531 |
|  |  | rs76531781 | -0.232 | 0.049 | 22.073 |
|  |  | rs9864379 | -0.139 | 0.027 | 26.858 |

Table S3 The heterogeneity results from the Cochran's Q test.

| Group | Gut microbiota | method | Cochran's Q | df | *P*-value |
| --- | --- | --- | --- | --- | --- |
| class | *Bacteroidia* | MR Egger | 15.956 | 13 | 0.251 |
|  |  | Inverse variance weighted | 18.239 | 14 | 0.196 |
| genus | *Blautia* | MR Egger | 19.007 | 11 | 0.061 |
|  |  | Inverse variance weighted | 19.033 | 12 | 0.088 |
| genus | *Parabacteroides* | MR Egger | 3.806 | 7 | 0.802 |
|  |  | Inverse variance weighted | 4546 | 8 | 0.805 |
| phylum | *Cyanobacteria* | MR Egger | 3.379 | 7 | 0.848 |
|  |  | Inverse variance weighted | 3.614 | 8 | 0.890 |
| order | *Bacteroidales* | MR Egger | 15.956 | 13 | 0.251 |
|  |  | Inverse variance weighted | 18.239 | 14 | 0.196 |
| genus | *Veillonella* | MR Egger | 5.472 | 7 | 0.603 |
|  |  | Inverse variance weighted | 7.154 | 8 | 0.520 |

Table S4 Directional pleiotropy results from Egger intercept analysis.

| Group | Gut microbiota | Egger intercept | SE | *P*-value |
| --- | --- | --- | --- | --- |
| class | *Bacteroidia* | -0.00029 | 0.000212 | 0.196 |
| genus | *Blautia* | -3.11E-05 | 0.000252 | 0.904 |
| genus | *Parabacteroides* | 0.000261 | 0.000303 | 0.418 |
| phylum | *Cyanobacteria* | -0.00017 | 0.000341 | 0.642 |
| order | *Bacteroidales* | -0.00029 | 0.000212 | 0.196 |
| genus | *Veillonella* | -0.00058 | 0.000446 | 0.236 |
