# Supplementary figures and images for: Causal associations between gut microbiota and cutaneous melanoma: a Mendelian randomization study
Source: Front Microbiol. 2024 Apr 8;15:1339621. doi: 10.3389/fmicb.2024.1339621 (PMC11033470; doi:10.3389/fmicb.2024.1339621)

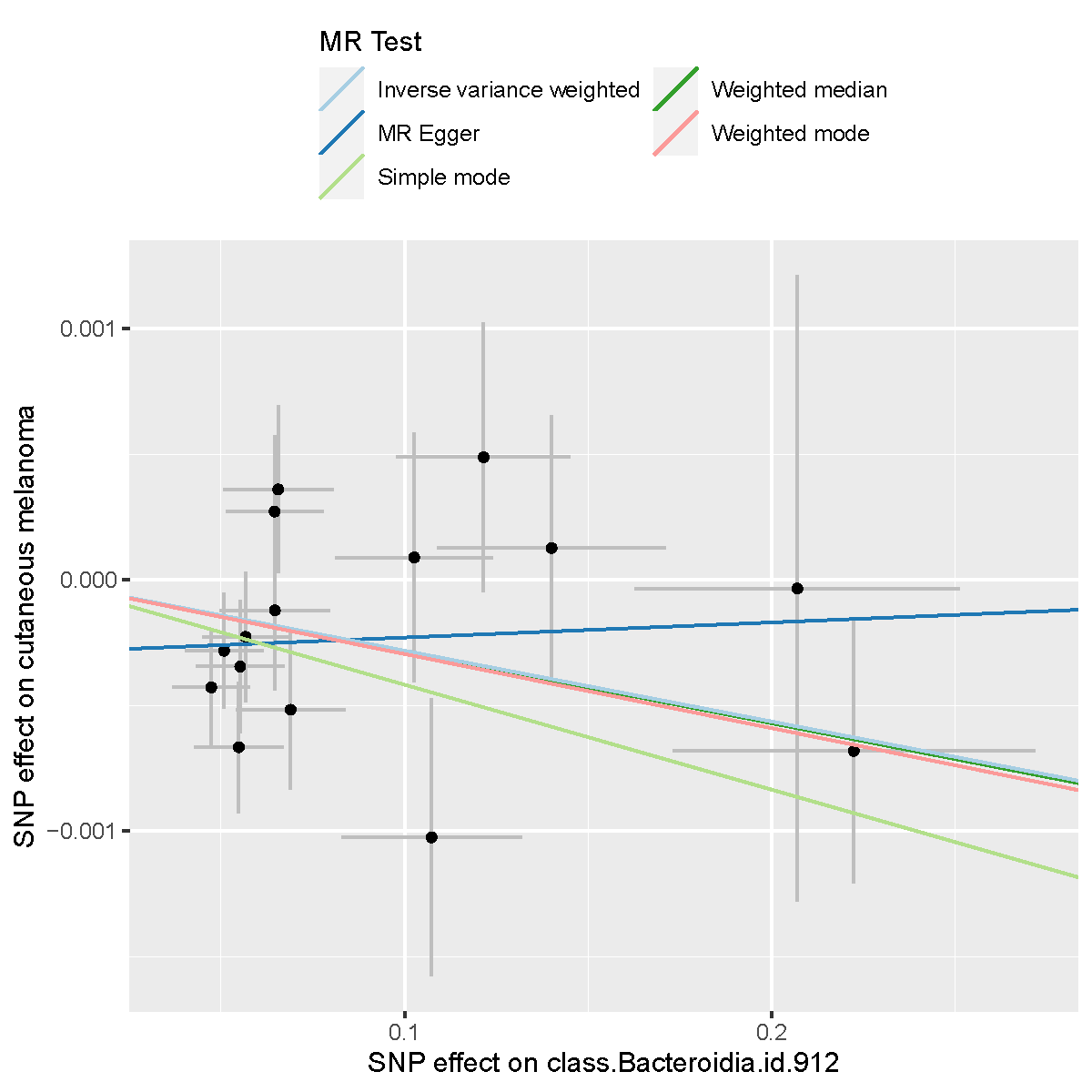

Supplement: Supplementary file 2 [file Data_Sheet_1.ZIP › Figure 2A.tif]

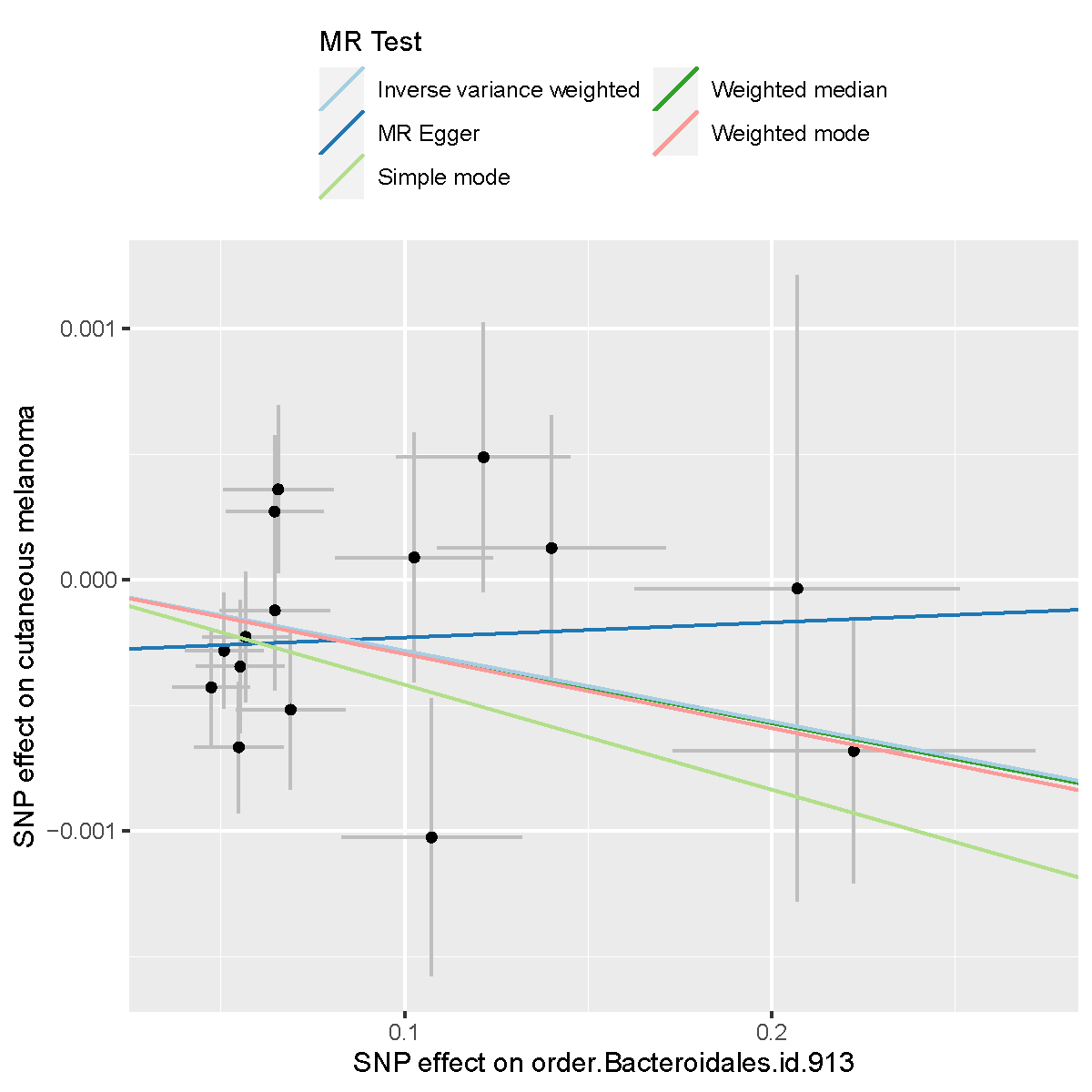

Supplement: Supplementary file 2 [file Data_Sheet_1.ZIP › Figure 2B.tiff]

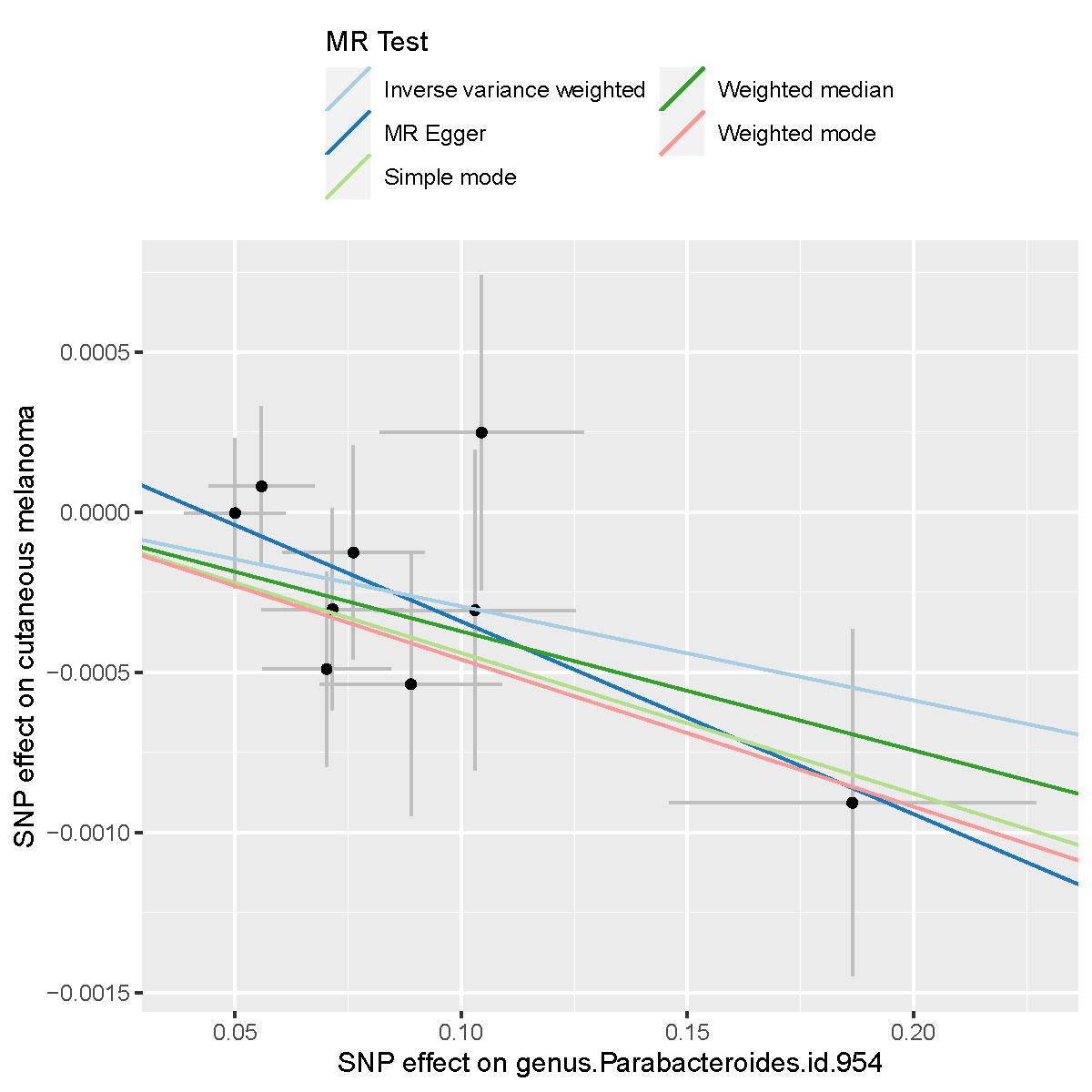

Supplement: Supplementary file 2 [file Data_Sheet_1.ZIP › Figure 2C.tif]

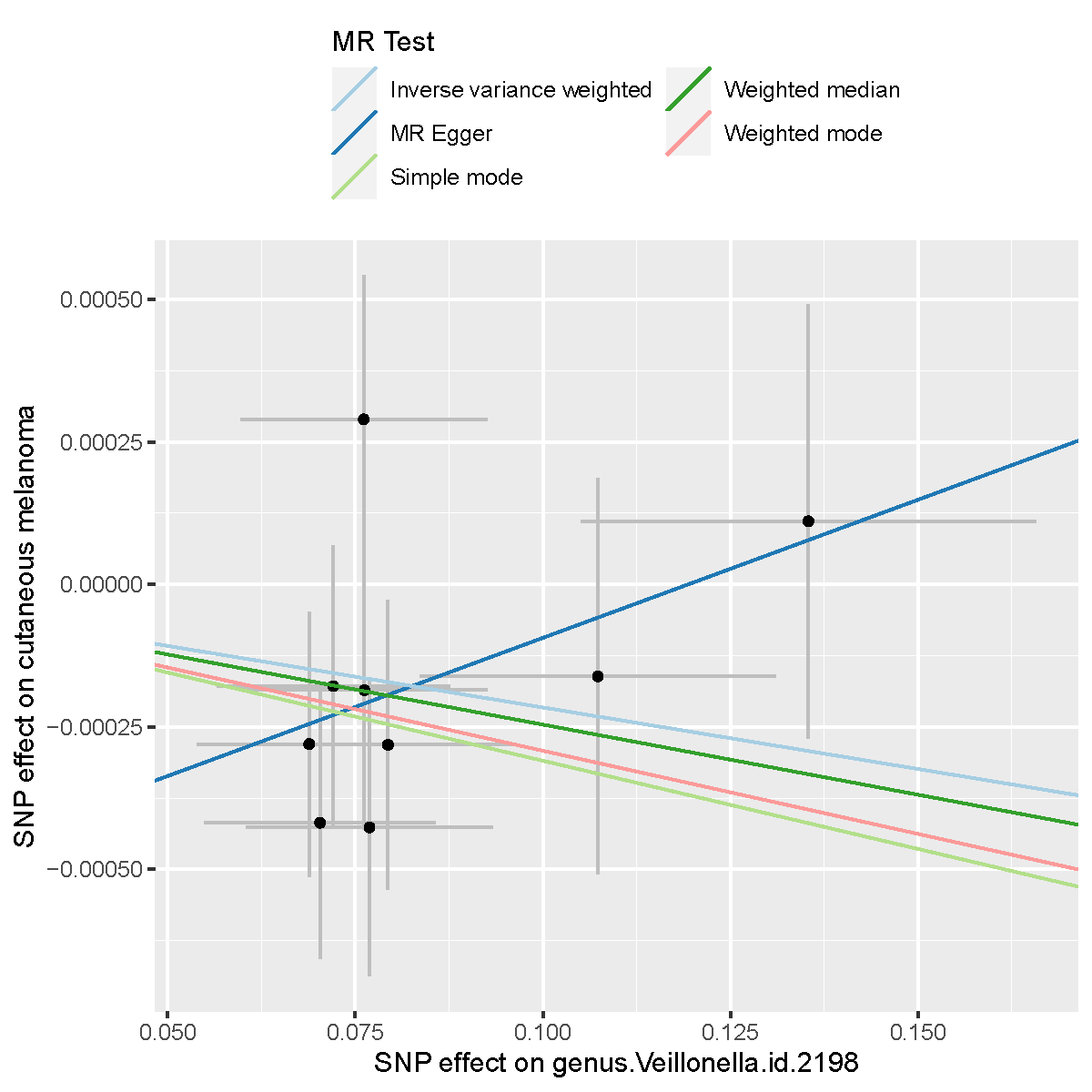

Supplement: Supplementary file 2 [file Data_Sheet_1.ZIP › Figure 2D.tif]

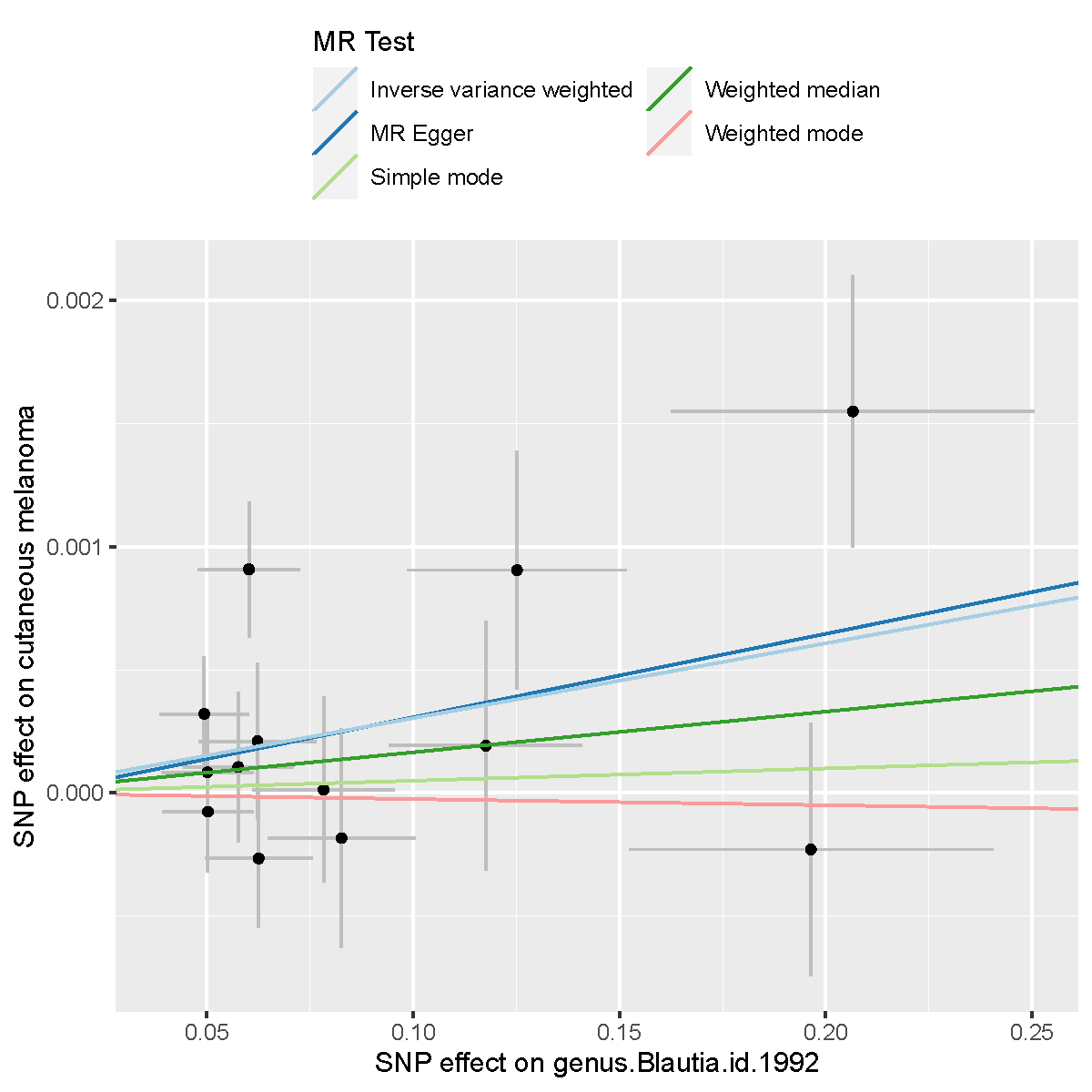

Supplement: Supplementary file 2 [file Data_Sheet_1.ZIP › Figure 2E.tif]

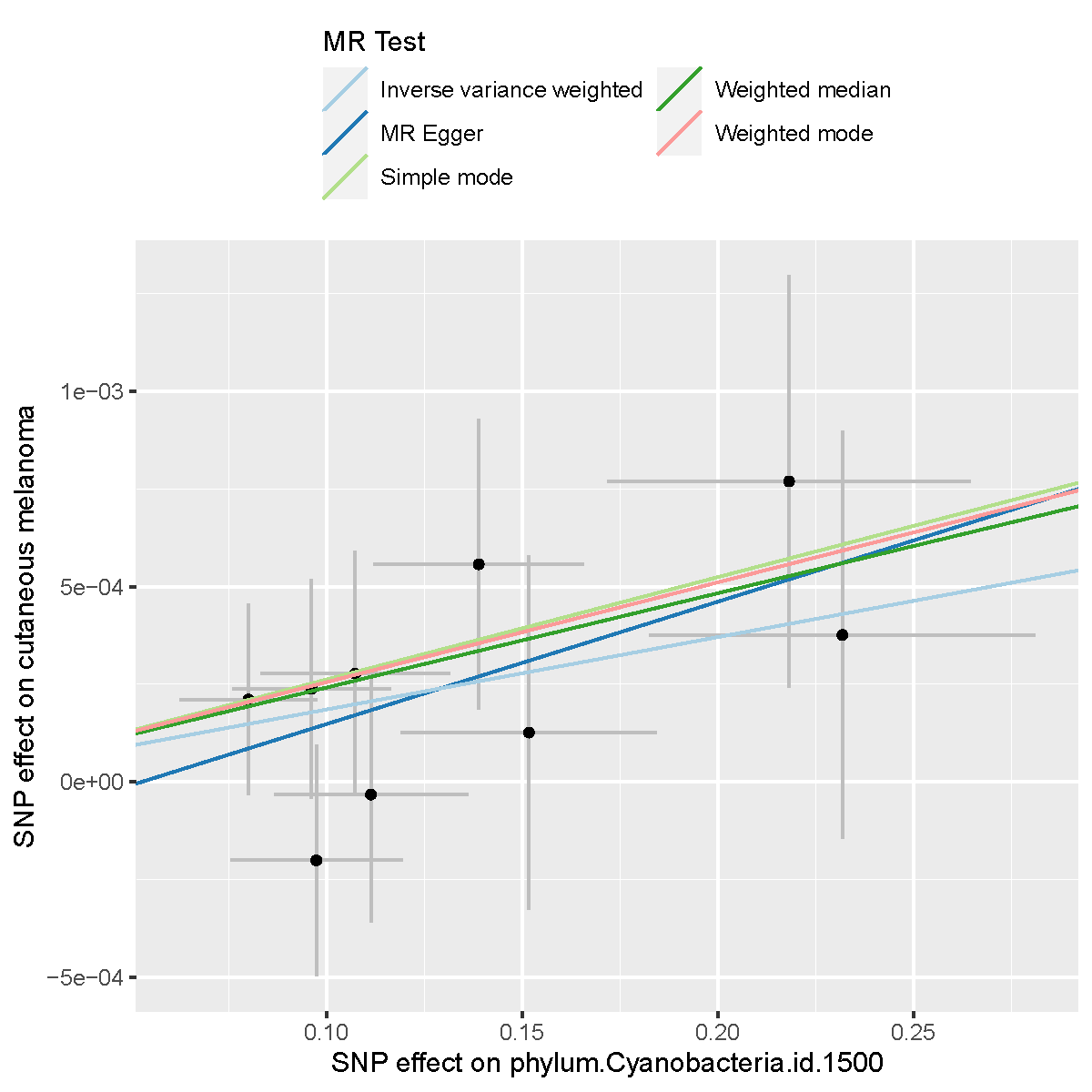

Supplement: Supplementary file 2 [file Data_Sheet_1.ZIP › Figure 2F.tif]

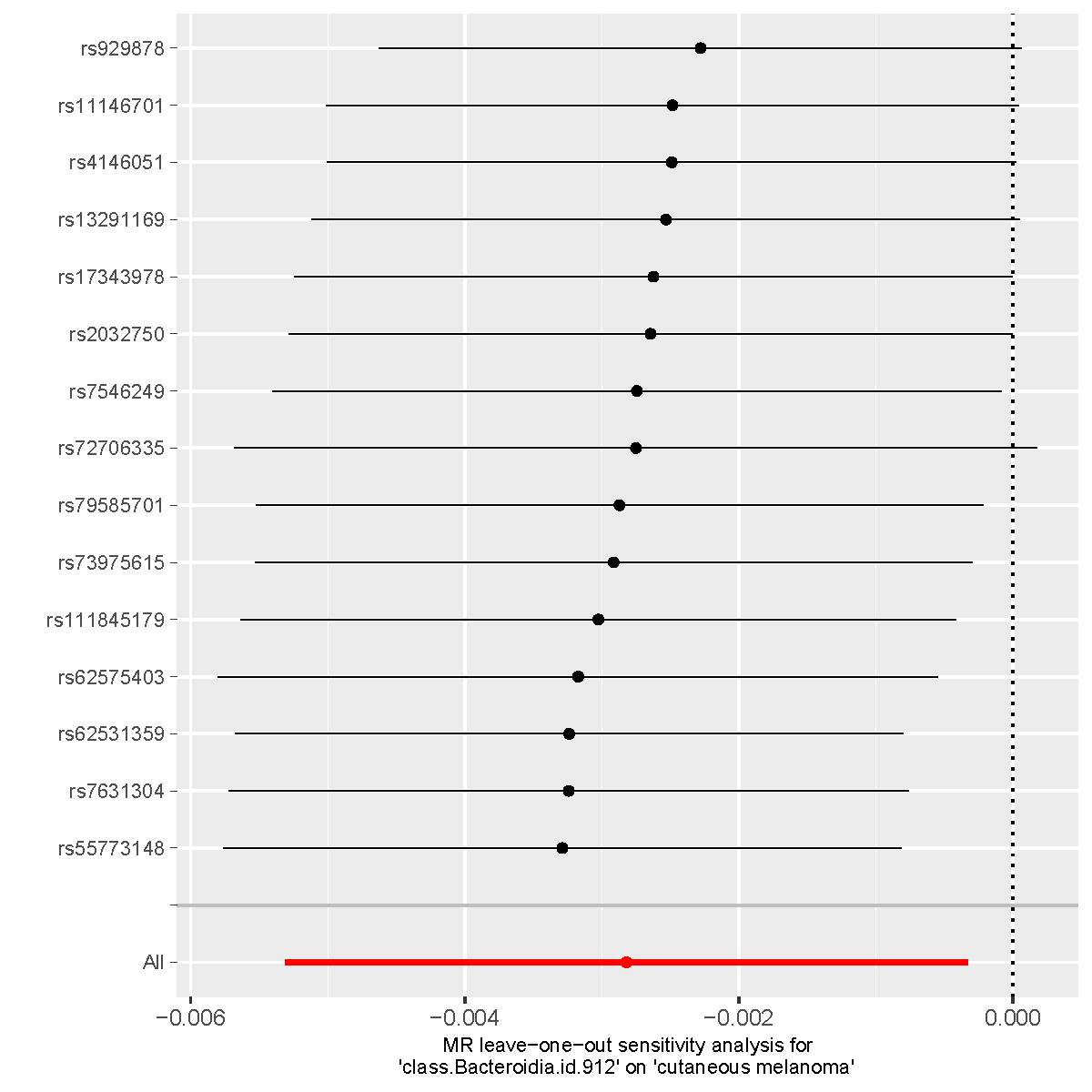

Supplement: Supplementary file 2 [file Data_Sheet_1.ZIP › Figure 3A.tif]

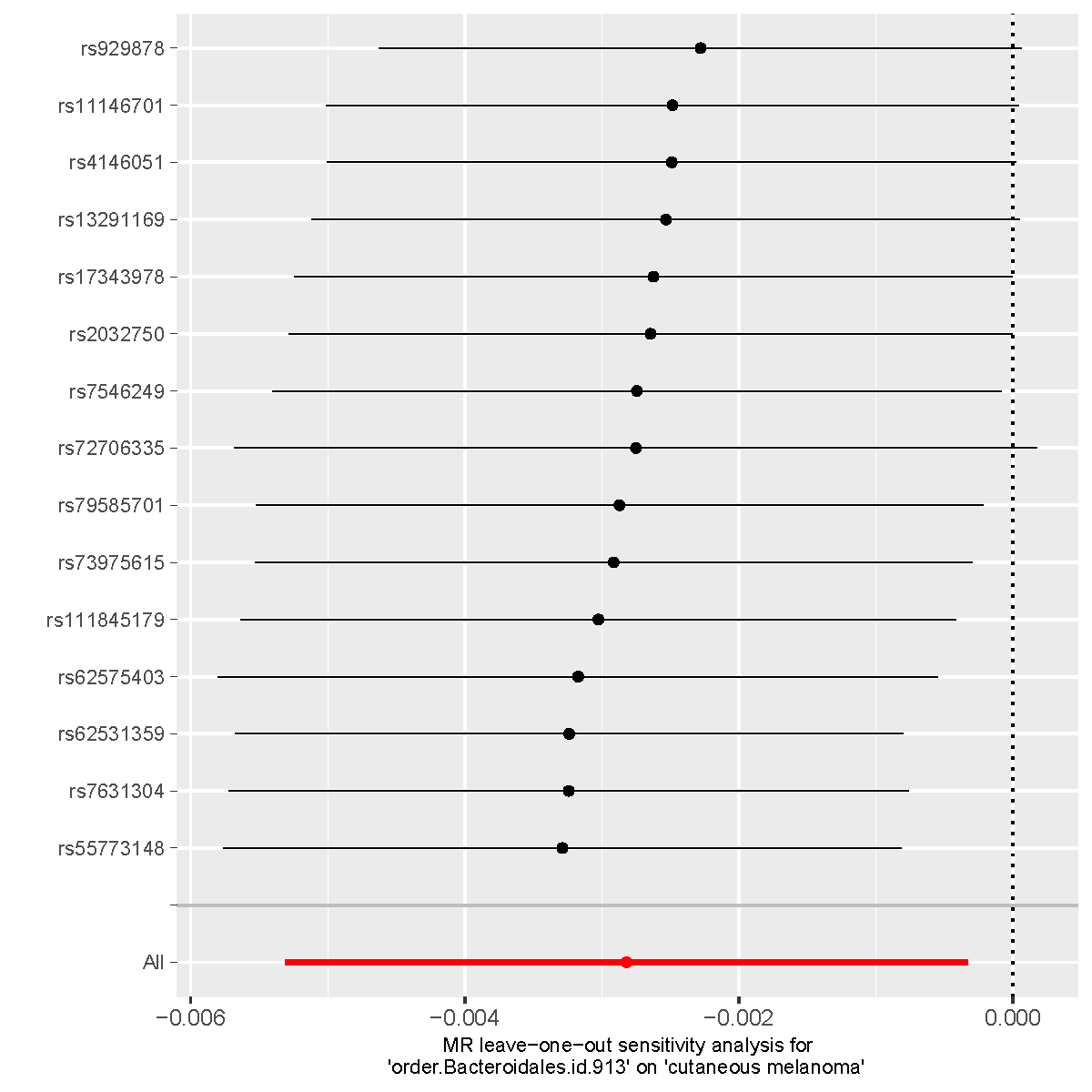

Supplement: Supplementary file 2 [file Data_Sheet_1.ZIP › Figure 3B.tiff]

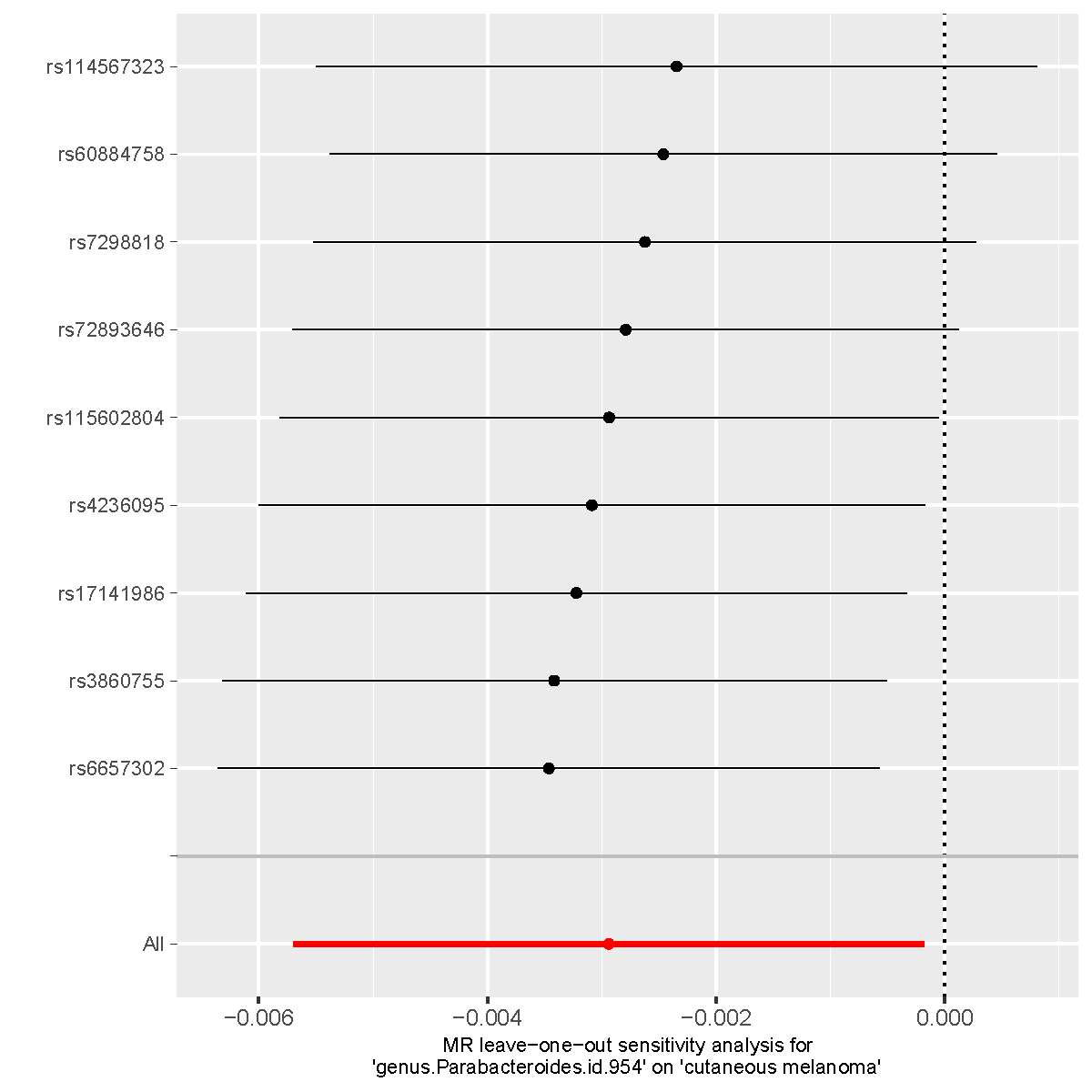

Supplement: Supplementary file 2 [file Data_Sheet_1.ZIP › Figure 3C.tif]

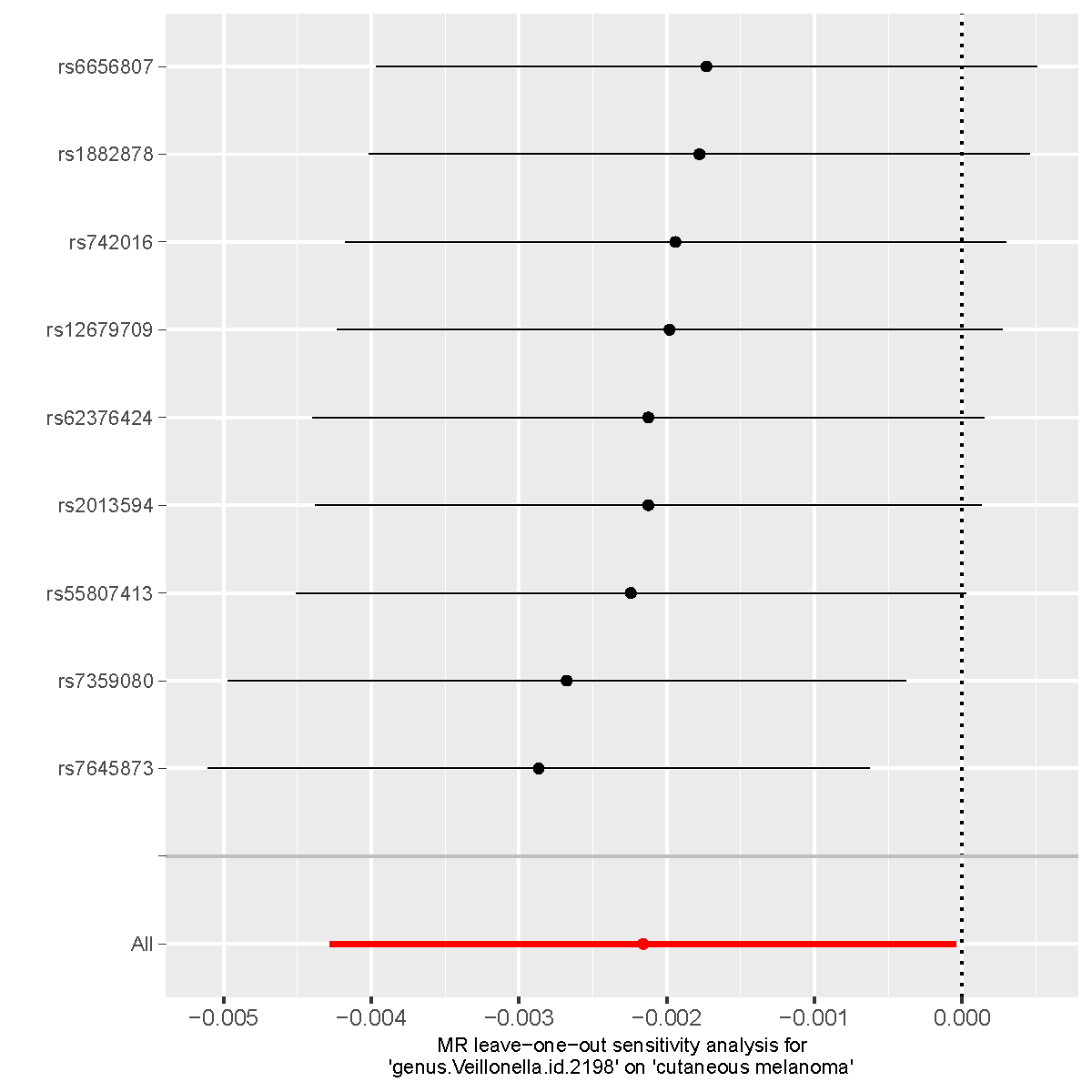

Supplement: Supplementary file 2 [file Data_Sheet_1.ZIP › Figure 3D.tif]

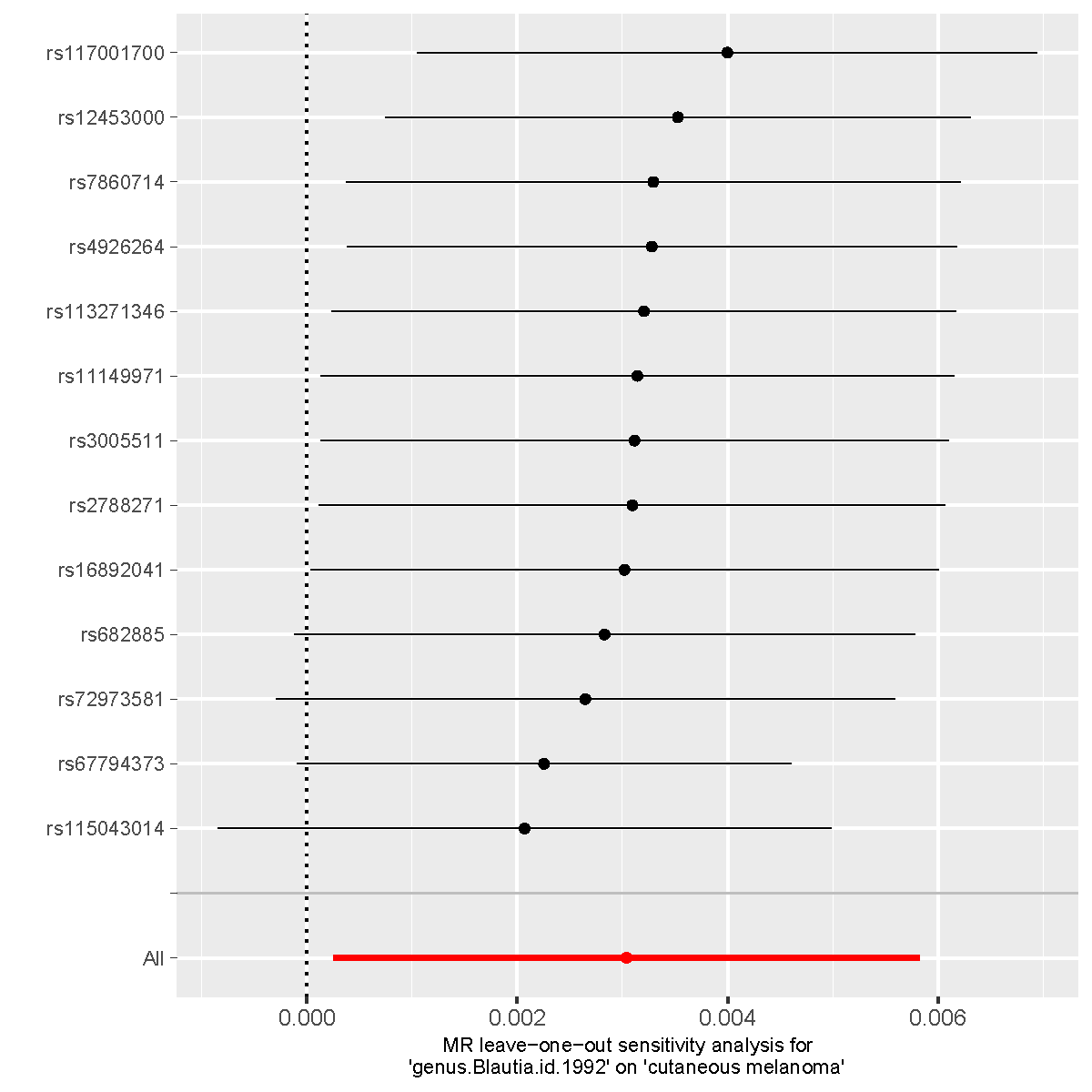

Supplement: Supplementary file 2 [file Data_Sheet_1.ZIP › Figure 3E.tif]

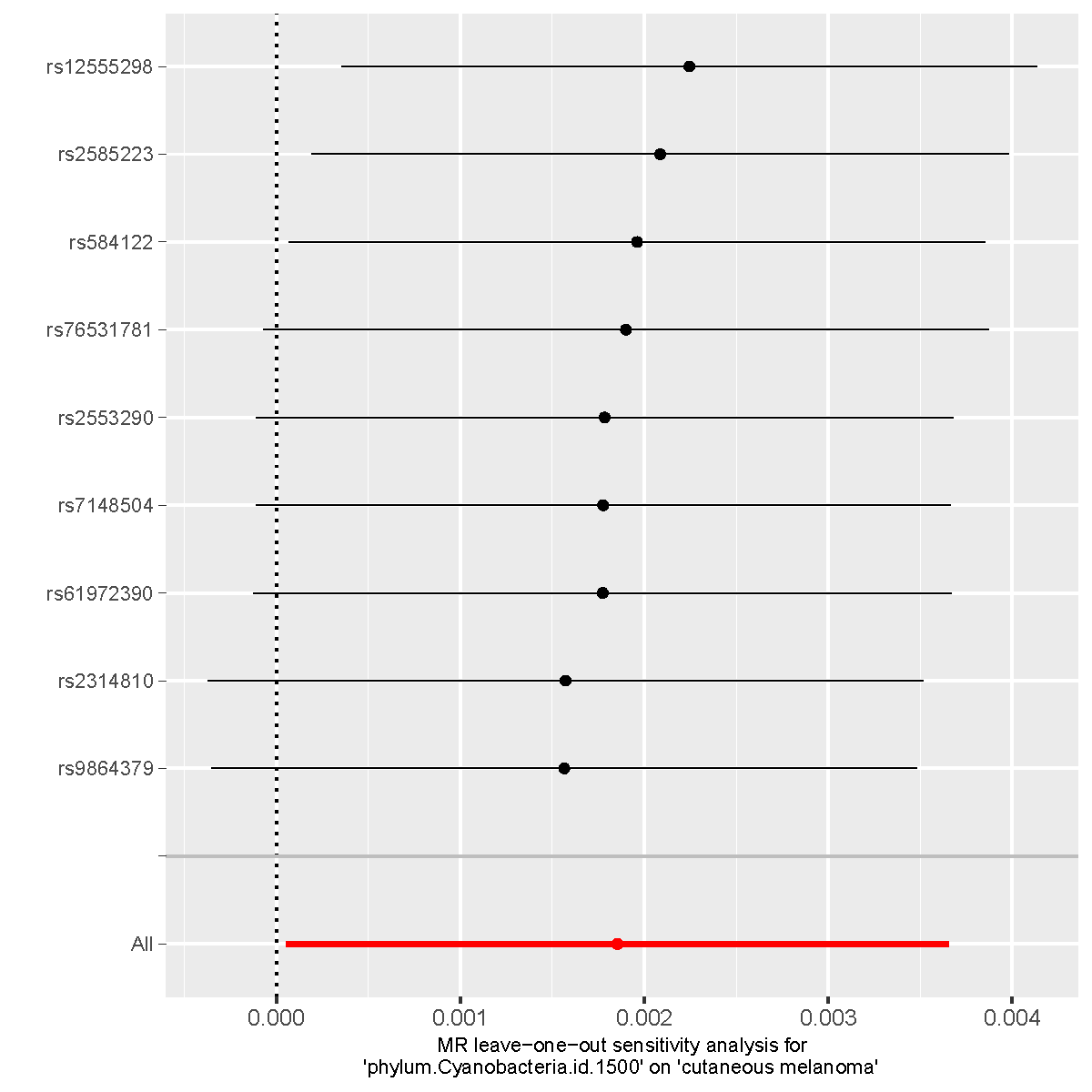

Supplement: Supplementary file 2 [file Data_Sheet_1.ZIP › Figure 3F.tif]
